# Integrative Analysis of Gut Microbiota and Metabolome Reveals Health Benefits of Postbiotic Supplementation in Asian Seabass (*Lates calcarifer*)

**DOI:** 10.3390/biology15181605

**Published:** 2026-09-11

**Authors:** Kuo-Chin Huang, Rolissa Ballantyne, Noppasorn Chumpati, Jannisa Punchanokkul, Yi-Liang Tu, Hsiao-Tung Chang, Jin-Seng Lin, Jai-Wei Lee, Phunsin Kantha, Chun-Hung Liu

**Affiliations:** 1 Department of Tropical Agriculture and International Cooperation, National Pingtung University of Science and Technology, Pingtung 912301, Taiwan; akimhuang@gmail.com (K.-C.H.); rolissa12@gmail.com (R.B.); joeylee@mail.npust.edu.tw (J.-W.L.); 2 Faculty of Fisheries, Kasetsart University, Bangkok 10900, Thailand; pondconan18@gmail.com (N.C.); jannisatip27@gmail.com (J.P.); phunsin.k@ku.th (P.K.); 3 Culture Collection & Research Institute, SYNBIOTEC Inc., Kaohsiung 82151, Taiwan; vanessa.tu@synbiotech.com.tw (Y.-L.T.); hsiaotung@synbiotech.com.tw (H.-T.C.); jslin@synbiotech.com.tw (J.-S.L.); 4 Department of Aquaculture, National Pingtung University of Science and Technology, Pingtung 912301, Taiwan; 5 National Museum of Marine Biology and Aquarium, Pingtung 94450, Taiwan

**Keywords:** postbiotic, Asian seabass, innate immunity, disease resistance, microbiome, metabolomics

## Abstract

This study evaluated the effects of dietary SYNSEA Premium postbiotics on growth, immunity, disease resistance, intestinal microbiota, and host metabolism in Asian seabass (*Lates calcarifer*). Fish were fed a control diet or diets supplemented with heat-killed *Lactiplantibacillus plantarum* LP28, *L. plantarum* LP1008, and *Bacillus subtilis* at 10^8^ (LSP) or 10^9^ (HSP) cells kg^−1^ diet for 56 days. Postbiotic supplementation did not significantly affect growth performance, feed efficiency, production, condition factor, or dorsal muscle composition, but significantly improved survival. Fish receiving postbiotics also exhibited higher survival following *Vibrio alginolyticus* and iridovirus challenges. These protective effects were accompanied by enhanced superoxide dismutase, phagocytic, and lysozyme activities and modulation of immune-related genes, including *tgf-β1*, *tnf*, *ifn-γ1*, *c3*, and *mx*. Exploratory microbiome and metabolome analyses, which were restricted to the control and LSP groups, identified differences in the relative abundance of specific intestinal microbial taxa and associations between microbial composition and host metabolic profiles. The LSP group showed lower relative abundances of potential pathogens such as *Salmonella enterica*, *Lactococcus garvieae*, and *Staphylococcus warneri*, although the overall microbial community structure did not differ significantly between groups. Metabolomic analysis of the LSP group further showed changes in D-glucose, pentose phosphate pathway intermediates, reduced glutathione, CoA, and 2-methylacetoacetyl-CoA relative to the control. Collectively, SYNSEA Premium improved survival, immune responses, and resistance to bacterial and viral infections without significantly affecting growth performance, while exploratory omics analysis of the LSP treatment identified associated microbial and metabolic changes.

## Simple Summary

Postbiotics are preparations made from inactivated beneficial microorganisms and their components, which may help improve animal health without using live bacteria. This study examined whether adding a commercial postbiotic product, SYNSEA Premium, to the diet of Asian seabass could affect growth, survival, immune function, resistance to disease, gut bacterial composition, and metabolism. Fish were fed diets with or without the postbiotic for 56 days and were then exposed to a disease-causing bacterium or virus. The postbiotic did not improve growth or feed use, but it increased fish survival and improved resistance to both bacterial and viral infections. It also enhanced several natural immune responses, reduced the abundance of some potentially harmful gut bacteria, and altered metabolic processes related to energy use and antioxidant protection. These findings suggest that dietary SYNSEA Premium can strengthen the overall health and disease resistance of Asian seabass even without promoting faster growth. Such an approach may help reduce losses from disease and support more sustainable and reliable aquaculture production.

## 1. Introduction

Asian seabass (*Lates calcarifer*), also known as barramundi, is one of the most commercially important finfish species in Indo-West Pacific aquaculture, including Taiwan, due to its rapid growth, high market value, and broad tolerance to salinity, which makes it suitable for diverse farming systems. Food and Agriculture Organization statistics indicate that the annual global production of Asian seabass is approximately 105,000 metric tons, with production increasing at an average rate of about 5% per year [1]. Despite its economic significance, seabass farming is frequently constrained by infectious diseases such as viral nervous necrosis, streptococcosis, and vibriosis, which can cause severe mortality and substantial economic losses [2,3,4]. These ongoing health challenges highlight the urgent need for improved management strategies and sustainable nutritional interventions. In addition, concerns regarding antimicrobial resistance and the sustainability of antibiotic-based disease control have increased the demand for effective non-antibiotic alternatives in aquaculture. Accordingly, functional feed additives, including probiotics and postbiotics, have gained increasing attention as promising approaches to enhance immune function, promote gut health, and improve disease resistance in Asian seabass aquaculture [5,6].

Probiotics and postbiotics have emerged as promising strategies for modulating gut microbial communities and enhancing intestinal health in aquaculture species [7]. According to the consensus statement of the International Scientific Association for Probiotics and Prebiotics (ISAPP), postbiotics are defined as preparations of inanimate microorganisms and/or their cellular components that confer measurable health benefits to the host [8]. These preparations comprise a diverse array of bioactive microbial-derived compounds, including cell wall constituents, metabolites, and extracellular factors, which have been shown to modulate host immune responses [9] and inhibit pathogen proliferation [10]. Despite the demonstrated benefits of live probiotics, their practical application in aquafeeds depends on maintaining sufficient microbial viability during feed processing, storage, and administration. Exposure to high temperature, pressure, and prolonged storage may reduce viable cell numbers and consequently affect product consistency. Postbiotics do not require maintenance of microbial viability and are therefore generally more stable during feed manufacture and storage. This characteristic may facilitate their incorporation into commercial aquafeeds and provide more consistent delivery than live probiotic preparations. Consequently, postbiotics are increasingly regarded as a practical alternative to live probiotics for functional aquafeed development [11,12].

Postbiotics may influence host physiology through microbial cellular components and other constituents retained in the preparation, with reported effects on immune responses, intestinal function, and disease resistance in aquatic animals [13]. Within aquaculture, these properties position postbiotics as a compelling alternative to conventional disease management strategies, including vaccination and chemotherapeutic interventions, although the underlying molecular mechanisms remain incompletely understood and require further elucidation. Emerging evidence has demonstrated that dietary incorporation of postbiotics can significantly improve growth performance in commercially important species, such as hybrid sturgeon (*Acipenser baerii* × *Acipenser schrenckii*) [14]. In addition to growth enhancement, postbiotics contribute to multiple aspects of host physiology, including modulation of gut microbial communities, improvement of metabolic efficiency, and reduction in susceptibility to infectious diseases [13,15,16]. Notably, application-specific studies further substantiate these benefits; for example, supplementation with postbiotics derived from *Bacillus subtilis* in Asian seabass has been shown to enhance immune competence, elevate antioxidant capacity, and confer increased resistance to infection by *Streptococcus iniae* [6]. However, current evidence in Asian seabass has largely focused on individual physiological responses or single-pathogen challenge models. Whether dietary postbiotics can simultaneously enhance resistance to both bacterial and viral pathogens remains poorly understood. Moreover, integrated studies combining gut microbiome and host metabolome analyses to evaluate these responses in Asian seabass are still scarce. Collectively, these knowledge gaps highlight the need for a more comprehensive evaluation of postbiotic-associated immune, microbial, metabolic, and disease-resistance responses in this species.

Therefore, the present study aimed to evaluate the effects of dietary SYNSEA Premium supplementation in juvenile Asian seabass. The study addressed three major aspects: (1) growth performance; (2) resistance to *Vibrio alginolyticus* and iridovirus infection, together with associated innate immune responses; and (3) intestinal microbial composition and host metabolic profiles in an exploratory comparison between the control and LSP groups, including integration of microbiome and metabolome data.

## 2. Materials and Methods

### 2.1. Source of the Postbiotic

The postbiotic products (SYNSEA Premium), formulated at two different concentrations with the same bacterial composition, were provided by SYNBIO TECH Inc. (Kaohsiung, Taiwan). The product consisted of heat-killed *Lactiplantibacillus plantarum* LP28, *L. plantarum* LP1008, *Pediococcus pentosaceus* PP49 and *B. subtilis*. Prior to heat inactivation, the bacterial cultures were diluted with maltodextrin to obtain two formulations with concentrations of 10^7^ and 10^8^ colony-forming unit (CFU) g^−1^, respectively. The resulting bacterial powders were confirmed to contain no viable bacterial cells. These were subsequently designated as low-strength postbiotics (LSP postbiotic) and high-strength postbiotics (HSP postbiotic).

### 2.2. Experimental Fish and Acclimation

Juvenile Asian seabass were obtained from a commercial fish farm and transported to the experimental facility. Upon arrival, the fish were acclimated in a 10-ton cement tank (6 m × 2 m × 1 m) supplied with continuous flow-through freshwater and aeration to maintain optimal water quality, with dissolved oxygen levels above 5 mg L^−1^. During the 7-day acclimation period, the fish were fed the control diet to apparent satiation twice daily until they were stable and fully adapted to the experimental conditions. Water temperature was maintained at 27–29 °C throughout the acclimation period. All animal experiments were conducted following protocols authorized by the Institutional Animal Care and Use Committee (IACUC) at the National Pingtung University of Science and Technology (NPUST). The study was approved under IACUC permit number NPUST-109-103, dated 2 February 2020.

### 2.3. Preparation of Experimental Diets

The ingredient composition and proximate analysis of the basal diet are provided in Table 1. All dry ingredients were first finely ground and passed through a sieve to obtain a uniform particle size. They were then weighed according to the proportions listed in Table 1 for the preparation of the experimental diets. For the LSP and HSP diets, 10 g kg^−1^ of the respective LSP postbiotic or HSP postbiotic preparation was added to achieve final postbiotic concentrations of 10^8^ cells kg^−1^ (LSP diet) and 10^9^ cells kg^−1^ (HSP diet), based on the labeled bacterial concentrations prior to heat inactivation. In the control diet, 10 g kg^−1^ of maltodextrin was added in place of the postbiotic preparations. All additives were premixed with a small portion of the basal mash prior to incorporation into the full batch to ensure homogeneous distribution. After dry mixing, the oil phase was gradually incorporated, followed by the addition of water to obtain a suitable consistency for pelleting. The dough was then pelleted using a laboratory-scale pellet mill, with the die size selected to match the mouth gape of the juveniles. The pellets were dried at 80 °C until the moisture content was reduced to below 10%. After cooling to room temperature, the diets were sealed in airtight bags and stored at 4 °C until use. Proximate compositions of the experimental diets were determined following methods of the Association of Official Analytical Chemists [17], including crude protein, crude lipids, and moisture.

### 2.4. Experimental Design and Growth Trial

The feeding trial was conducted at the aquaculture farm of National Pingtung University of Science and Technology (Pingtung, Taiwan) for a period of 56 days. During the growth trial, juvenile Asian seabass (initial body weight: 5.9 ± 0.2 g) were randomly distributed into 9 floating cages (1.0 m × 0.4 m × 0.4 m), which were installed inside 2.5-ton cement tanks (1.2 m × 1.9 m × 1.2 m). A total of three tanks were used, with three cages installed in each tank. Each dietary treatment was represented by one cage in each tank, resulting in three replicate cages per treatment. Fish were randomly allocated to the replicate cages, with 50 fish stocked in each cage. Each floating cage was equipped with a screen (pore size: approximately 120 μm) at the bottom to collect uneaten feed. The tanks were supplied with continuous overflow water at a flow rate of approximately 20 L h^−1^ and aeration to maintain dissolved oxygen levels above 5 mg L^−1^ throughout the experimental period. Fish were fed the assigned diets twice daily at a feeding rate of 5% of body weight. Uneaten feed was siphoned from the bottom of each cage after feeding, immediately dried at 80 °C, and weighed to calculate feed intake. Fish were group-weighed at 14-day intervals until the end of the experiment. Water quality parameters, including ammonia-N and nitrite-N, were measured biweekly according to the methods described by Bower et al. [18] and Bendschneider et al. [19], respectively. At the end of the feeding trial, fish were group-weighed to determine survival, weight gain, feed efficiency, condition factor, and total production per cage. These parameters were calculated using the following equations:

Survival (%) = (final number of fish/initial number of fish) × 100%;

Percentage of weight gain (%) = ((FW − IW)/IW) × 100%;

Feed efficiency = weight gain/total feed intake;

Protein efficiency ratio (PER) = weight gain (g)/protein intake (g);

Protein intake (g) = total feed intake (g) × dietary crude protein content.

Condition factor = (weight/Length^3^) × 100;

Total production per cage (g) = average body weight × number of surviving fish.

where IW and FW represent the initial and final body weights, respectively.

Following the growth trial, fish were assessed for health status, including immune responses, expression of immune-related genes, and resistance to bacterial and viral pathogens, as well as gut microbiota composition and metabolic status of the dorsal muscle and liver.

### 2.5. Evaluation of the Health Status of Fish

Healthy fish were randomly selected from each treatment group for immunological assessments and pathogen challenge tests. For the evaluation of immune parameters, two fish were sampled from each of the three replicate tanks, resulting in a total of six fish per treatment group. Similarly, two fish per replicate tank were collected for the analysis of immune-related gene expression, giving six fish per treatment group. An additional six fish per treatment group, with two fish sampled from each replicate tank, were intraperitoneally injected with nervous necrosis virus (NNV) for subsequent evaluation of mx gene expression.

For the pathogen challenge tests, ten fish were randomly selected from each replicate tank, resulting in a total of thirty fish per treatment group for the bacterial challenge. Another thirty fish per treatment group, likewise consisting of ten fish from each of the three replicate tanks, were used for the viral challenge experiment. Fish were therefore sampled evenly across the replicate tanks within each treatment to maintain balanced representation and avoid pseudo-replication.

#### 2.5.1. Challenge Tests

For the bacterial challenge assay, *V. alginolyticus* [20] was cultured in tryptic soy broth (TSB; Gibco, MD, USA) at 28 °C for 24 h. The bacterial cells were then collected by centrifugation at 7000× *g* for 10 min at 4 °C, washed, and resuspended in sterile 0.85% NaCl solution to obtain the stock suspension. The challenge experiment was conducted in triplicate, involving a total of 30 fish per dietary group (10 fish per replicate). Each fish was intraperitoneally injected with 50 µL of the bacterial suspension, delivering a dose of 4 × 10^6^ CFU g^−1^ body weight (Median Lethal Dose, LD_50_). The LD_50_ dose used in the bacterial challenge was determined based on a preliminary experiment conducted prior to the formal challenge test. An additional 30 fish from the control group were intraperitoneally injected with 50 µL of sterile saline and served as unchallenged controls. Following injection, fish were transferred to 80-L glass aquaria (10 fish per tank) containing 60 L of freshwater maintained at 28 °C. Throughout the challenge period, the rearing water was replaced daily, and mortality was monitored and recorded for each treatment group. Fish mortality was determined when individuals showed no response to physical stimulation and opercular movement had ceased. Observation was discontinued when no fish mortality was observed for two consecutive days and the fish resumed feeding.

For the iridovirus challenge assay, fish were individually captured using a hand net and immediately administered an intraperitoneal injection of 100 µL iridovirus [21] suspension at a concentration equivalent to 5 × 10^7^ TCID_50_ g^−1^ body weight (LD_50_). The LD_50_ dose used in the viral challenge was also determined based on a preliminary experiment conducted prior to the formal challenge test. Each dietary treatment consisted of three replicate tanks, with 10 fish allocated to each 60-L glass aquarium filled with 40 L of freshwater. An additional 30 fish from the control group were intraperitoneally injected with 100 µL of sterile saline and served as unchallenged controls. Continuous aeration was provided in all tanks, and approximately 50% of the water was replaced daily to ensure suitable water quality. Throughout the experimental period, fish were inspected at least twice per day for clinical symptoms, mortality, and alterations in feeding behavior. Fish were considered dead when they failed to respond to physical stimulation and no opercular movement was observed. The challenge experiment was concluded once no mortalities were recorded for two successive days and normal feeding activity had fully recovered.

#### 2.5.2. Immune Parameters

Fish immune parameters were analyzed according to the methods described by Huang et al. [20]. Fish were anaesthetized by immersion in ice water. Blood samples were then collected from the caudal vein using a 24-gauge needle and a 3-mL syringe for serum preparation. Head kidney leukocytes were subsequently isolated from the head kidney tissue using density gradient centrifugation with a discontinuous Percoll gradient (34%/51%) (P1644, Sigma, St. Louis, MO, USA). After separation, cell viability was assessed using the trypan blue exclusion method. Viable leukocytes were suspended in Leibovitz’s L-15 medium (L4386, Sigma, St. Louis, MO, USA) and adjusted to a final concentration of 106 cells mL^−1^ for subsequent immune parameter assays.

Respiratory burst activity was quantified using a microplate-based nitroblue tetrazolium (NBT) reduction assay. This colorimetric method measures the production of reactive oxygen species (ROS) generated during the respiratory burst of leukocytes, which results in the intracellular reduction in NBT to insoluble dark blue formazan deposits.

Phagocytic activity of head kidney leukocytes was evaluated using a fluorescence-based assay in 12-well culture plates. Fluorescent latex beads (L4655, Sigma, St. Louis, MO, USA) were used as inert phagocytic targets in place of FITC-labeled pathogens. Leukocytes were incubated with the fluorescent beads for 1 h at room temperature to allow phagocytosis. After incubation, non-ingested beads were removed by washing, and the cells were fixed with 2% paraformaldehyde for 1 h at 4 °C. Fixed cells were subsequently stained with 0.1% propidium iodide and washed three times with phosphate-buffered saline (PBS). Phagocytic activity was determined by examining the samples under a fluorescence microscope, and results are expressed as the percentage of phagocytic leukocytes according to the following formula:

Phagocytic activity = (phagocytic leukocytes/total leukocytes counted) × 100%.

Head kidney leukocytes were suspended in PBS (pH 7.8) and centrifuged at 300× *g*. The pellet was washed twice with PBS, resuspended in 100 μL PBS, and homogenized using an ultrasonic homogenizer. The homogenate was then centrifuged at 10,000× *g* for 30 min at 4 °C, and the supernatant was collected as head kidney leukocyte lysate (HLS). Ten microliters of HLS was thoroughly mixed with 0.05 M PBS, 13 mM methionine, 0.1 mM Na_2_EDTA, 63 μM NBT, and 1.5 μM vitamin B12 solution, followed by incubation at room temperature for 10 min. Subsequently, 200 µL of the reaction mixture was transferred to a 96-well microplate, and absorbance was measured at 560 nm using a microplate spectrophotometer (SpectraMax^®^ 190, Sunnyvale, CA, USA) [22]. A unit of superoxide dismutase (SOD) is defined as the amount of enzyme that causes 50% inhibition of NBT reduction. SOD activity is expressed as units per gram of protein (U (g protein)^−1^).

Blood samples were allowed to clot at room temperature for 30 min, and then stored at 4 °C overnight. Serum was obtained by centrifugation at 4 °C for 5 min and used for lysozyme activity analysis. Serum lysozyme activity was determined using a turbidimetric assay with 0.02% *Micrococcus lysodeikticus* (M3770, Sigma, St. Louis, MO, USA) as the substrate. Briefly, serum samples were incubated with a suspension of *M. lysodeikticus* prepared in 0.05 M sodium phosphate buffer (pH 6.2) at 25 °C. Changes in absorbance were recorded at 530 nm after 1 and 6 min using a UV–visible spectrophotometer (SpectraMax^®^ 190, Molecular Devices, Sunnyvale, CA, USA). Lysozyme activity was quantified based on a calibration curve generated with hen egg white lysozyme (L6876, Sigma, St. Louis, MO, USA) and is expressed relative to protein content. Serum protein concentration was measured using the Bio-Rad Protein Assay (Bio-Rad Laboratories, Inc., Hercules, CA, USA) with bovine serum albumin as the reference standard.

#### 2.5.3. Immune-Related Gene Expressions

Immune-related genes, including mechanistic target of rapamycin (*mtor*), mammalian lethal with SEC13 protein 8 (*mlst-8*), eukaryotic translation initiation factor 4E (*eif4e*), C-reactive protein (*crp*), complement components C3 (*c3*) and C4 (*c4*), heat shock cognate 70 kDa protein (*hsp70*), heat shock cognate 90 kDa protein (*hsp90*), transforming growth factor beta 1 (*tgf-β1*), tumor necrosis factor (*tnf*), interferon gamma 1 (*ifn-γ1*), interleukin-8 (*il-8*), and interleukin-10 (*il-10*), were analyzed [20]. *β-actin* was used as the internal reference gene. The primer sequences used in this study are listed in Table S1.

Fish were sampled and anesthetized by immersion in ice water. Following anesthesia, fish were dissected on ice, and the head kidney and liver tissues were collected for total ribonucleic acid (RNA) isolation. In addition, the expression of the *mx* gene, a key molecule involved in antiviral defense, was analyzed in the spleen following nervous necrosis virus (NNV) stimulation. Fish were individually injected with 20 μL of NNV at a dose of 10^4^ TCID_50_ per fish prior to RNA extraction. After viral injection, fish were transferred to floating cages in a cement tank under the same environmental conditions as those used during the growth trial. At 12 and 24 h post-injection, spleen tissues were individually sampled for total RNA isolation.

Total RNA from frozen tissues was extracted and purified using ULTRA-SPEC™ RNA Total RNA Isolation Reagent (Biotecx, Houston, TX, USA), followed by treatment with RQ1 RNase-Free DNase (Promega, Madison, WI, USA), according to the manufacturers’ protocols. For first-strand complementary DNA (cDNA) synthesis, 5 μg of total RNA from each tissue was reverse-transcribed using SuperScript II RNase H^−^ reverse transcriptase (Promega) with oligo(dT_18_) primers, following the manufacturer’s recommended reaction conditions. Gene expression analysis was performed using real-time polymerase chain reaction (qPCR) with SYBR Green chemistry (2× qPCRBIO SyGreen Blue Mix Hi-ROX, PCRBIOSYSTEMS, London, UK). Relative mRNA expression levels of target genes were normalized to the reference gene and calculated using the 2^−ΔΔCt^ method [23].

### 2.6. Gut Microbiota

At the conclusion of the 56-day feeding trial, intestinal microbial communities were characterized by next-generation sequencing (NGS) in fish fed the control and LSP diets. For microbial analysis, mid-intestinal samples were collected from three fish per dietary group, with one fish randomly selected from each replicate cage. The omics analysis was limited to the control and LSP groups and was intended to characterize microbiota-associated responses to LSP supplementation rather than to assess dose-dependent effects. The HSP group was not included in this analysis.

Total bacterial DNA was extracted from the mid-intestinal contents, and the full-length V1–V9 region of the 16S rRNA gene was amplified and sequenced by a commercial biotechnology service provider. The quality and size distribution of PCR amplicons were initially assessed using the D5000 ScreenTape Assay on an Agilent TapeStation 4200 system (Agilent Technologies, Waldbronn, Germany). Amplicons from all samples were then pooled and purified using SMRTbell^®^ cleanup beads (PacBio Biosciences, Menlo Park, CA, USA), followed by library construction with the SMRTbell^®^ Prep Kit 3.0 according to the manufacturer’s instructions. Barcoded single-molecule real-time (SMRT) libraries were subjected to an additional bead-based purification step, quantified using a Qubit 1X dsDNA High Sensitivity Assay Kit (Thermo Fisher Scientific, Waltham, MA, USA), and re-evaluated for fragment size distribution prior to sequencing. Sequencing was conducted on a PacBio Sequel IIe platform (PacBio Biosciences, Menlo Park, CA, USA). Raw subreads were processed into high-fidelity circular consensus sequences (CCSs) using the CCS algorithm implemented in SMRT Link software (version 25.3).

Taxonomic assignment of amplicon sequence variants (ASVs) was performed using the RDP Naive Bayesian Classifier [24] with a bootstrap confidence threshold of 50%, based on reference sequences from NCBI RefSeq BioProjects 33175 and 33317 [25], complemented by the Ribosomal Database Project (RDP) database [26]. Alpha-diversity metrics, including Shannon’s diversity index, Pielou’s evenness, Simpson index and Margalef’s species richness index, were calculated using QIIME 2. Beta-diversity was evaluated by principal coordinates analysis (PCoA) based on Bray–Curtis dissimilarity, performed on the Metware Cloud platform (Metware Biotechnology Co., Ltd., Wuhan, China). Patterns of species dominance and cumulative contributions of taxa were further examined using the PRIMER software package (version 6.1.5).

### 2.7. Metabolite Analysis

To investigate metabolic changes in the liver and dorsal muscle associated with postbiotic supplementation, an untargeted metabolomics approach was applied to fish from the control and LSP groups. As with the gut microbiota analysis, the HSP group was not included; therefore, the metabolomic analysis was not intended to assess dose-dependent responses. Tissue samples for metabolomic analysis were collected from the same three fish per group used for intestinal microbiota analysis, rapidly frozen in liquid nitrogen, and ground into powder using a pre-chilled mortar and pestle. About 50 mg of each powdered tissue was extracted with methanol:acetonitrile:ddH_2_O (2:2:1, *v*/*v*/*v*). The extracts were subjected to one freeze–thaw cycle and further homogenized using a tissue grinder. After centrifugation at 12,000 rpm for 15 min at 4 °C, the supernatants were obtained and used for metabolomic analysis.

Metabolite analysis was carried out using a Dionex UltiMate 3000 UPLC system coupled to an electrospray ionization source and a Q Exactive™ Plus Hybrid Quadrupole-Orbitrap™ mass spectrometer (Thermo Fisher Scientific, Waltham, MA, USA). The analytical procedure followed that reported by Cheng et al. [27]. Raw mass spectrometry data were processed with Progenesis QI software version 3.0 (Waters Corporation, Milford, MA, USA) for peak alignment, feature detection, and deconvolution. Metabolomic profiling was conducted using multivariate statistical analysis in MetaboAnalyst. Metabolites were initially assessed based on variable importance in projection (VIP), fold change (FC), and *p*-value (Student *t*-test; *p* < 0.05) criteria. Metabolites were specifically screened using a threshold of VIP > 1 combined with fold change values. This approach ensured that the metabolites selected represented those contributing most strongly to group discrimination and exhibiting biologically meaningful changes in abundance. Comparisons between the control and LSP groups were conducted separately for liver and muscle tissues. The identified differential metabolites were then annotated against the Kyoto Encyclopedia of Genes and Genomes (KEGG) database, followed by pathway enrichment analysis in MetaboAnalyst.

### 2.8. The Correlations Among Microbiota and Metabolites, and Construction of the Metabolite–Microbe Network

Potential associations between the intestinal microbiota and host metabolism were evaluated using Spearman’s rank correlation analysis. Microbial taxa detected in the intestinal samples of the control and LSP groups were included when their relative abundance exceeded 0.1%. These taxa were correlated with significantly altered metabolites identified in the muscle and liver based on their VIP scores and adjusted q-values. The relative abundance of each selected microbial taxon was correlated with the corresponding metabolite concentration. Statistical analyses were performed in R version 4.4.2, and correlation coefficients and *p*-values were calculated for each microbe–metabolite pair. Associations with an absolute correlation coefficient greater than 0.6 and a *p*-value below 0.05 were considered significant. Significant correlations were subsequently imported into Cytoscape version 3.10.3 to construct and visualize microbe–metabolite correlation networks. Network visualization was used to illustrate the patterns of significant associations among microbial taxa and metabolites.

### 2.9. Data Analysis

All statistical analyses were conducted with SAS version 9.4 (SAS Institute Inc., Cary, NC, USA). Before applying parametric tests, the normality of the data and equality of variances were examined using the Shapiro–Wilk and Levene’s tests, respectively. Data satisfying these assumptions were analyzed by one-way analysis of variance. When a significant treatment effect was detected, Tukey’s honestly significant difference test was applied for multiple comparisons among treatment means. Survival data obtained from the challenge trial were evaluated using Kaplan–Meier analysis, with differences among survival curves assessed by the log-rank test. Statistical significance was defined at *p* < 0.05.

## 3. Results

### 3.1. Growth Performance

The growth performance of fish fed the experimental diets for 56 days is presented in Table 2. Throughout the experimental period, water quality remained within acceptable ranges, with water temperature maintained at 27–29 °C and ammonia–N and nitrite–N concentrations at 0.01–0.03 mg L^−1^. These conditions indicate that fish growth was unlikely to have been adversely affected by water quality during the feeding trial. No significant differences were observed among dietary treatments in final body weight, percentage weight gain, feed efficiency, protein efficiency ratio, total production per tank, or condition factor. However, dietary treatment significantly affected survival. Fish fed the control diet showed the lowest survival rate, which was significantly lower than that of fish fed the postbiotic-supplemented diets, including both LSP and HSP. These results indicate that dietary supplementation with postbiotics did not significantly improve growth performance under the present experimental conditions, but it enhanced fish survival during the 56-day feeding trial. In addition, no significant differences were observed in dorsal muscle proximate composition (Table 3) or fatty acid profiles (Table S2) among the dietary treatments.

### 3.2. Bacterial and Viral Challenge Tests

**Figure 1:**
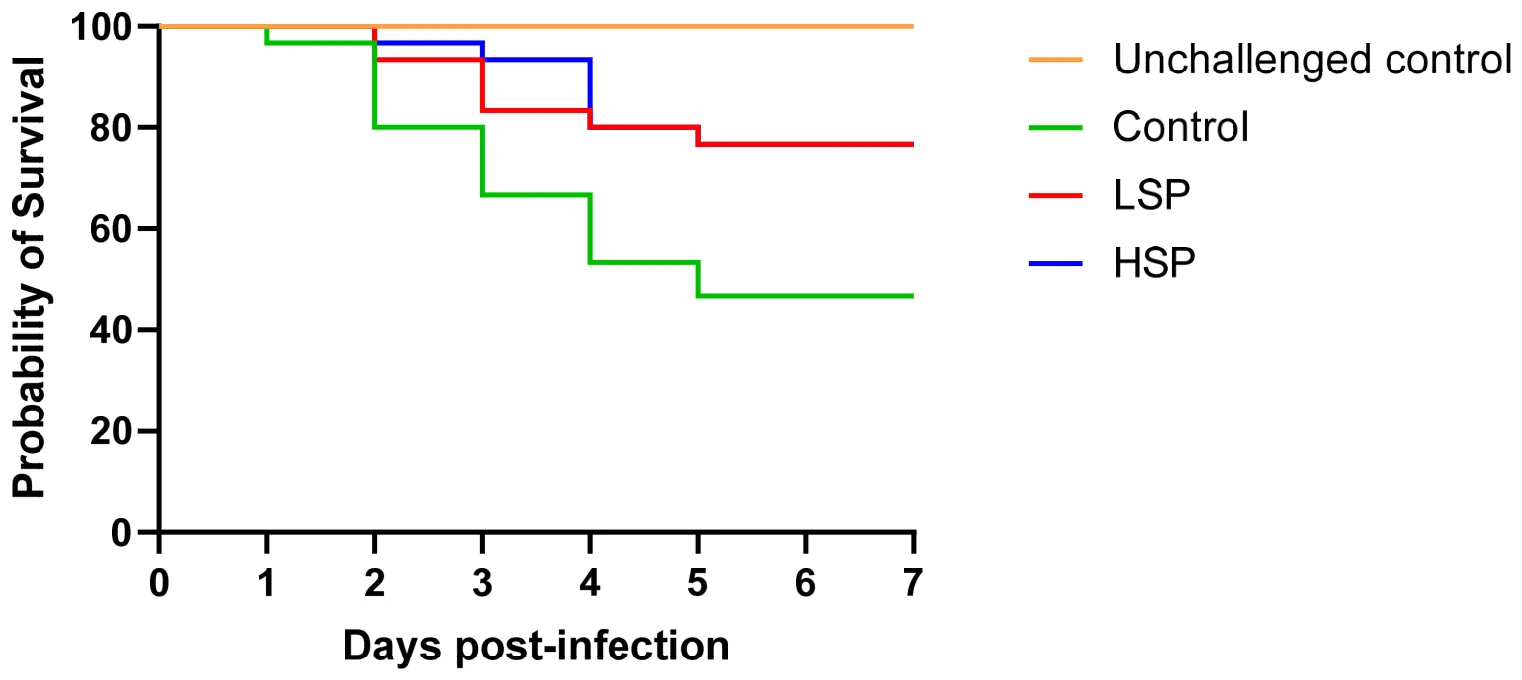
Kaplan–Meier survival curves of Asian seabass fed the control diet or diets supplemented with low- or high-dose postbiotics (LSP and HSP, respectively) for 56 days and subsequently challenged with *Vibrio alginolyticus* at 4 × 10^6^ CFU (g weight)^−1^. An unchallenged control group was included for comparison. The challenge experiment was conducted with three replicate tanks per treatment, with 10 fish in each tank. Survival was monitored for 7 days post-infection. Differences in survival distributions among groups were evaluated using the log-rank test (*p* < 0.05).

No mortality occurred in the unchallenged control groups during either challenge test. Following the bacterial challenge, mortality occurred earlier in fish fed the control diet than in those receiving the LSP or HSP diet. Survival subsequently declined in all challenged groups but stabilized toward the end of the observation period. Normal feeding activity also resumed before termination of the challenge in accordance with the predefined endpoint criteria. Kaplan–Meier analysis showed that fish fed either postbiotic-supplemented diet had significantly higher survival than those fed the control diet (Figure 1).

**Figure 2:**
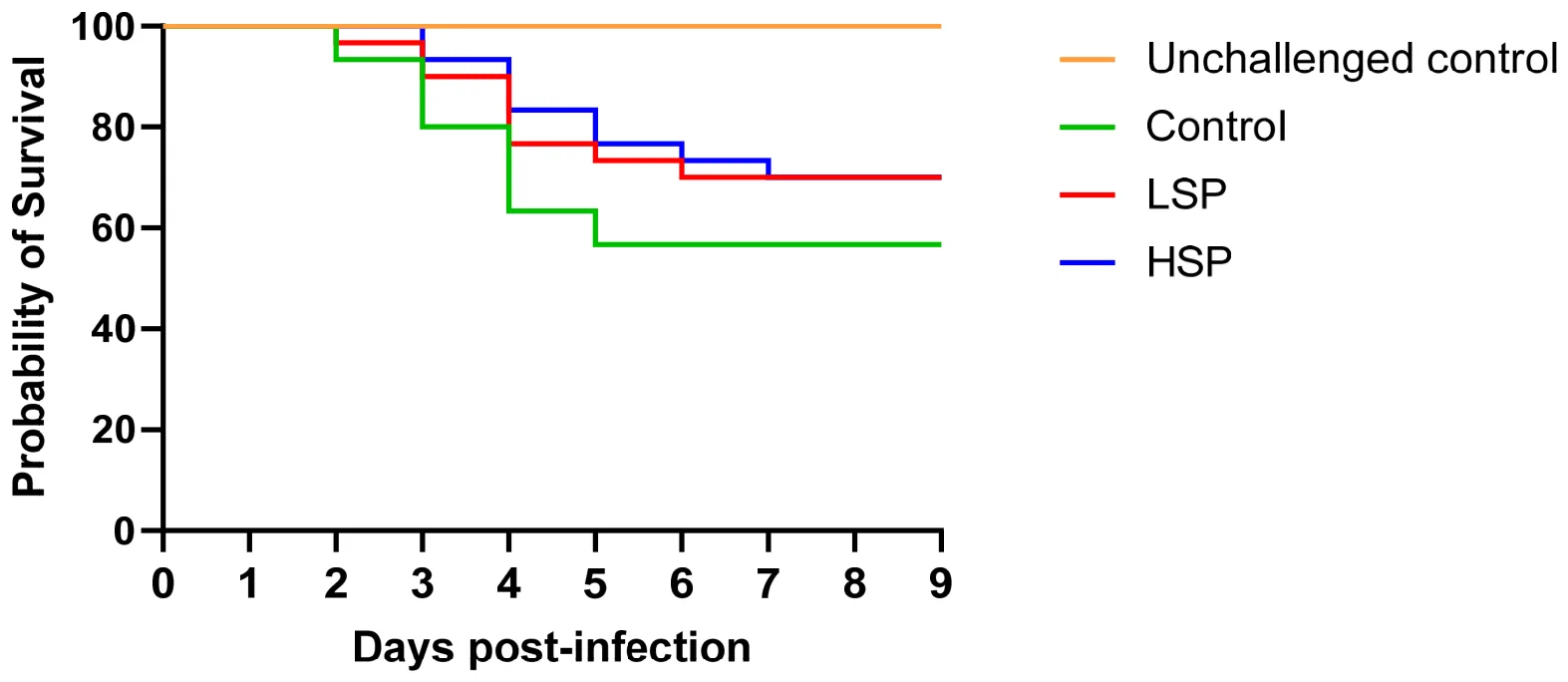
Kaplan–Meier survival curves of fish fed the control diet or diets supplemented with low- or high-dose postbiotics (LSP and HSP, respectively) for 56 days, followed by challenge with Iridovirus (5 × 10^7^ TCID (g weight)^−1^). Survival was monitored for 9 days post-infection. An unchallenged control group was included for comparison. The challenge experiment was conducted with three replicate tanks per treatment, with 10 fish in each tank. Differences in survival distributions among groups were evaluated using the log-rank test (*p* < 0.05).

In the viral challenge, survival remained at 100% in the unchallenged control group throughout the 9-day observation period. Mortality was first recorded on day 2 post-infection in the challenged control and LSP groups, whereas the first mortality in the HSP group occurred on day 3. Thereafter, survival declined progressively in all challenged groups. Kaplan–Meier survival analysis showed that fish fed the control diet had a significantly lower probability of survival than those fed the LSP and HSP diets at the end of the challenge period (Figure 2). Both postbiotic-supplemented groups maintained higher final survival rates than the challenged control group, indicating that dietary supplementation with SYNSEA Premium improved the resistance of Asian seabass to viral infection under the present experimental conditions.

### 3.3. Immune Responses

**Figure 3:**
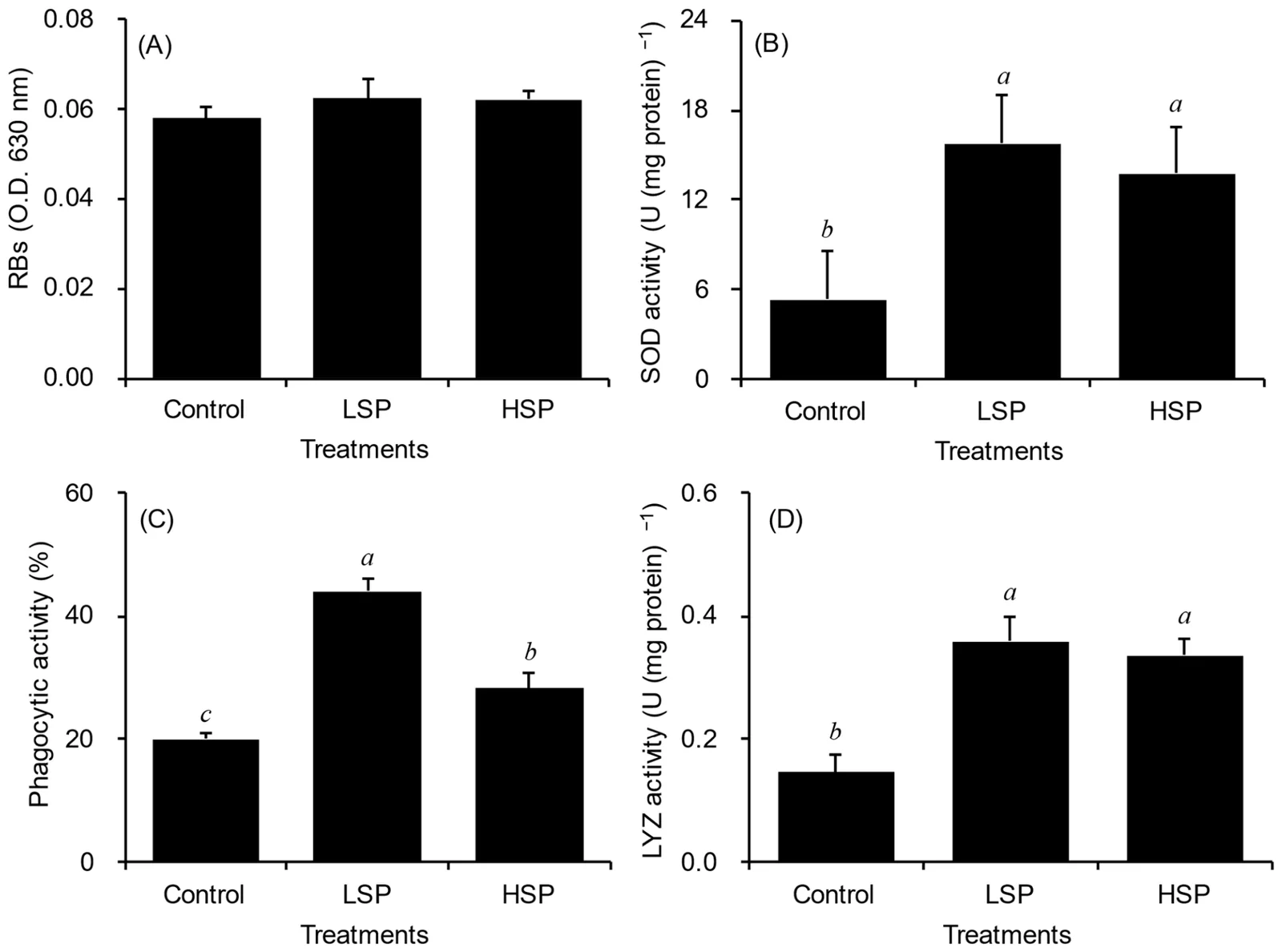
Respiratory bursts (RBs) (**A**), superoxide dismutase (SOD) activity (**B**), phagocytic activity (**C**), and lysozyme (LYZ) activity (**D**) of fish fed different experimental diets for 56 days. Data are presented as mean ± SE (*n* = 6), where fish were sampled evenly from different tanks. Different letters indicate significant differences among groups (*p* < 0.05).

No significant differences in respiratory burst activity were observed among the dietary treatments (Figure 3A; *p* > 0.05). In contrast, SOD activity was significantly higher in fish fed the LSP and HSP diets than in those fed the control diet (Figure 3B). Phagocytic activity also differed significantly among treatments (Figure 3C). Fish fed the postbiotic-supplemented diets, including both LSP and HSP, exhibited significantly higher phagocytic activity than those fed the control diet. Notably, fish fed the LSP diet showed significantly greater phagocytic activity than those fed the HSP diet. Lysozyme activity was significantly enhanced in fish fed the LSP and HSP diets compared with the control group (Figure 3D). These results suggest that dietary supplementation with postbiotics enhanced several innate immune responses in Asian seabass, particularly antioxidant capacity, phagocytic activity, and lysozyme activity, although respiratory burst activity was not significantly affected.

### 3.4. Immune-Related Genes

**Figure 4:**
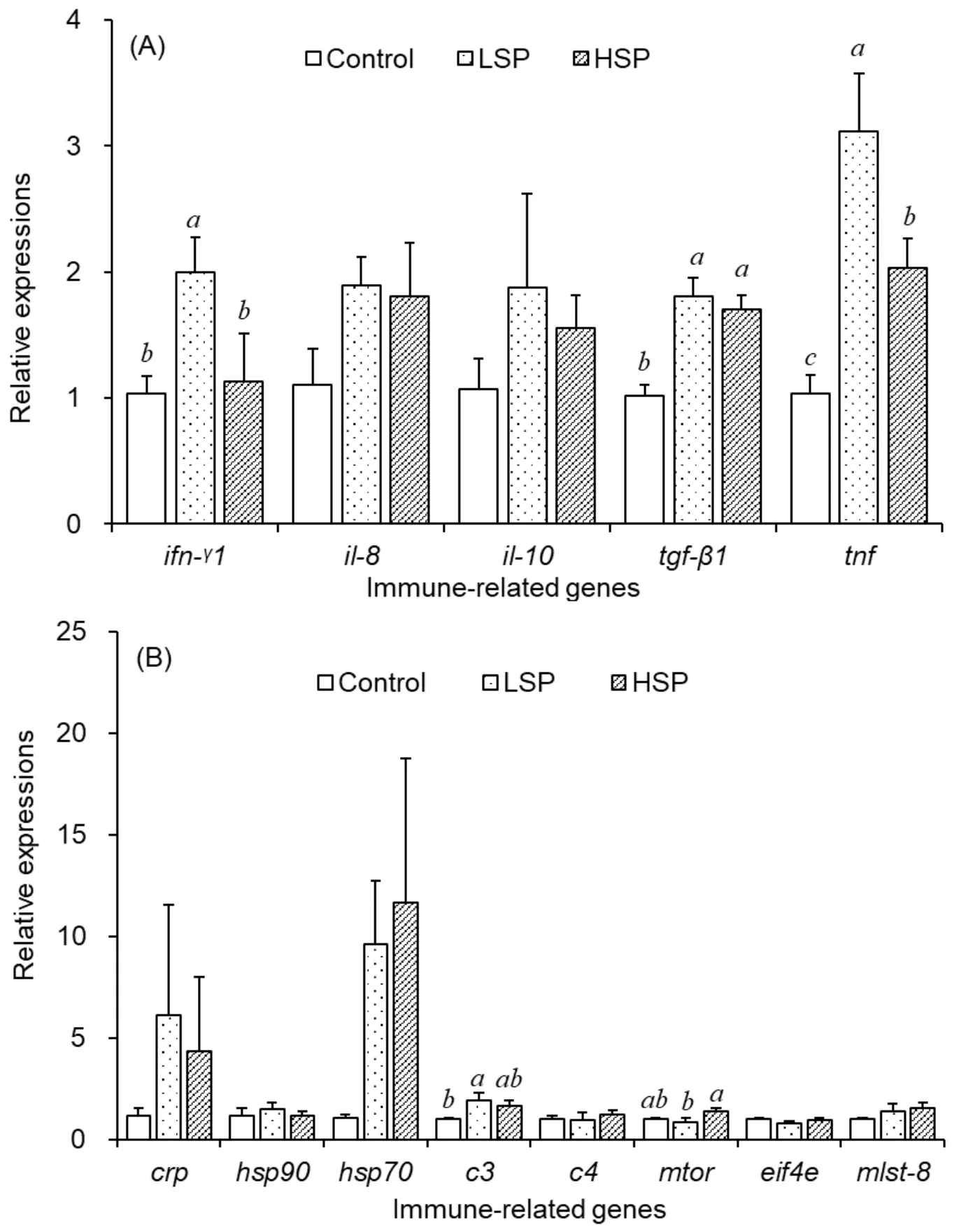
Relative expression of immune-related genes in the kidney (**A**) and liver (**B**) of fish fed different experimental diets for 56 days. Data are presented as mean ± SE (*n* = 6), where fish were sampled evenly from different tanks. Different letters indicate significant differences among groups (*p* < 0.05).

The expression levels of immune-related genes in fish fed the experimental diets for 56 days are presented in Figure 4A,B. No significant differences were observed among dietary treatments in the expression of *il-8*, *il-10*, *crp*, *hsp90*, *hsp70*, *c4*, *mtor*, *eif4e*, or *mlst-8* after 56 days of feeding (*p* > 0.05). In contrast, dietary treatment significantly affected the expression of several immune-related genes (*p* < 0.05). As shown in Figure 4A, the expression levels of *tgf-β1* and *tnf* were significantly upregulated in fish fed the postbiotic-supplemented diets, including both LSP and HSP, compared with the control group. The expression of *ifn-γ1* was significantly increased in fish fed the LSP diet compared with the control group, whereas no significant difference was observed between the HSP and control groups. As shown in Figure 4B, *c3* expression was significantly upregulated only in fish fed the LSP diet after 56 days of feeding, while no significant difference was detected between the control and HSP groups.

**Figure 5:**
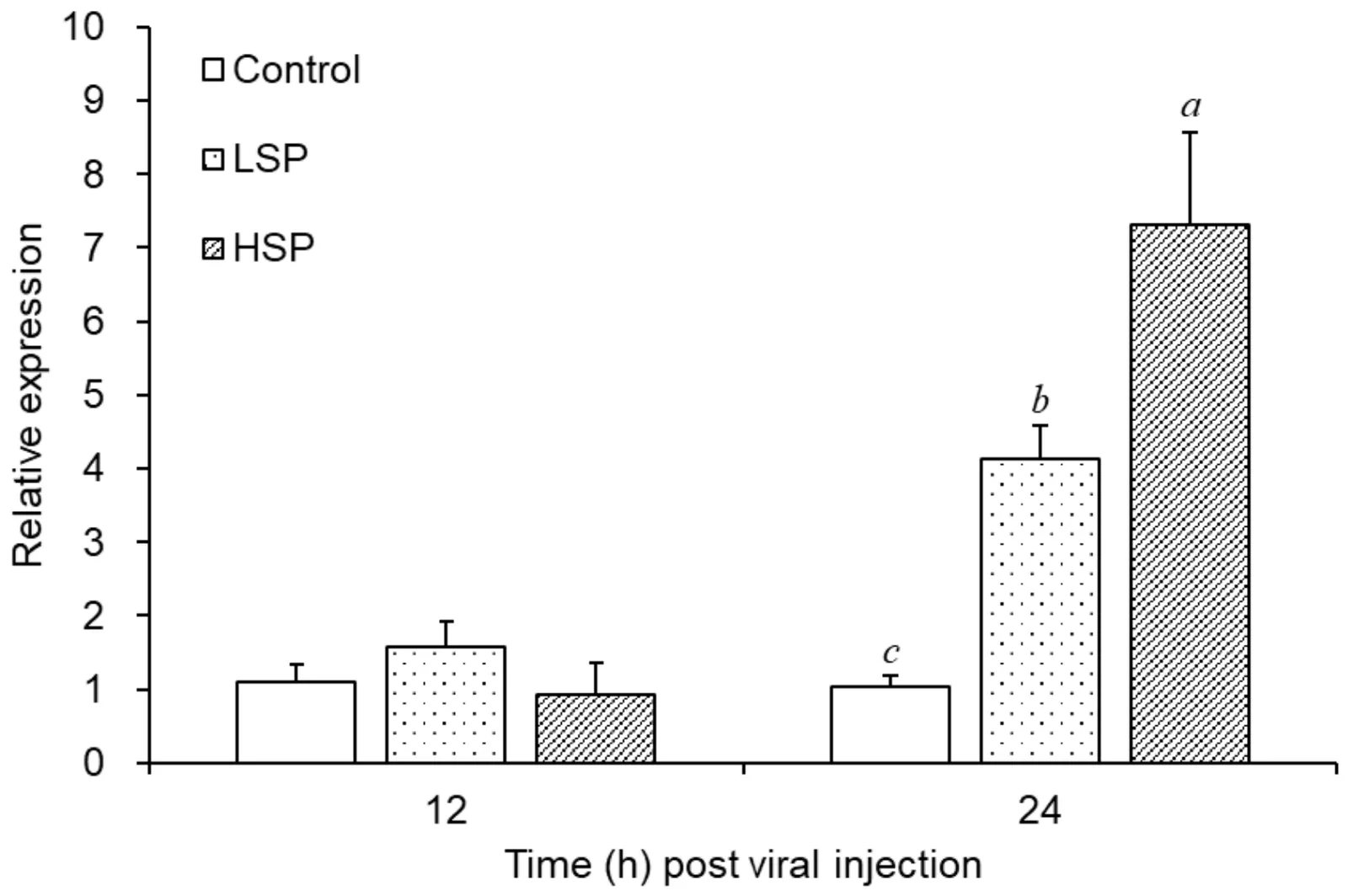
Relative expression of the *mx* gene in the spleen of fish fed the experimental diets for 56 days and subsequently challenged with Nervous Necrosis Virus (NNV) for 12 and 24 h. Data are presented as mean ± SE (*n* = 6), where fish were sampled evenly from different tanks. Different letters indicate significant differences among groups at the same time point (*p* < 0.05).

The relative expression of the *mx* gene in the spleen of Asian seabass after NNV challenge is shown in Figure 5. At 12 h post-viral injection, no significant differences in *mx* expression were observed among the control, LSP, and HSP groups (*p* > 0.05). However, at 24 h post-injection, *mx* expression was significantly affected by dietary treatment. Fish fed the LSP and HSP diets exhibited significantly higher *mx* expression than those fed the control diet, with the highest expression observed in the HSP group. Moreover, *mx* expression in the HSP group was significantly higher than that in the LSP group. These results indicate that dietary postbiotic supplementation increased splenic *mx* expression following NNV challenge.

### 3.5. Gut Microbiota

**Figure 6:**
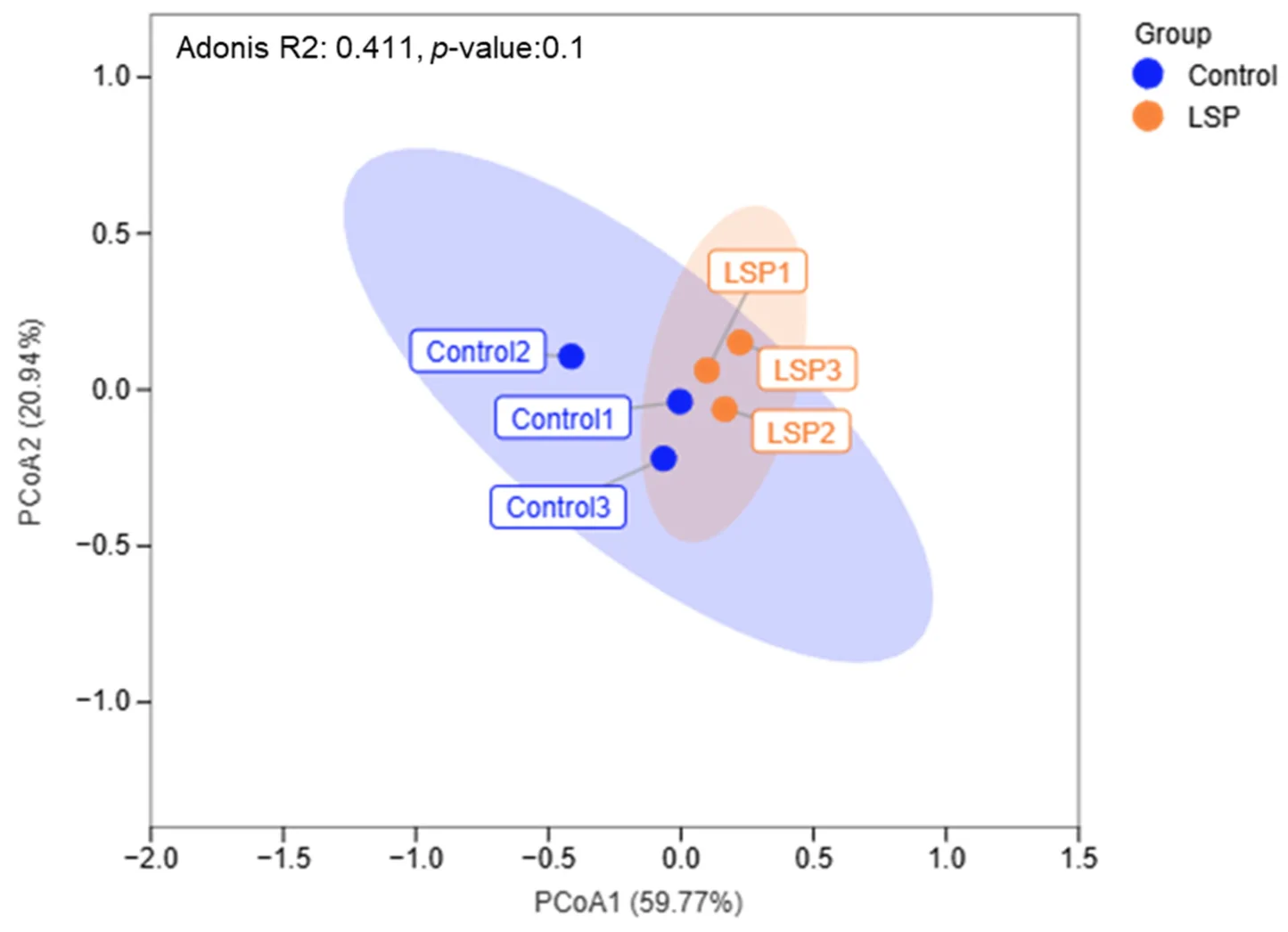
Principal coordinate analysis (PCoA) of intestinal microbial community composition in Asian seabass after 56 days of feeding with different diets. Samples from the control and postbiotic (LSP) groups are plotted based on Bray–Curtis dissimilarity. Each point represents an individual sample, and shaded ellipses indicate the 95% confidence intervals for each dietary group. The percentages on the axes represent the variation explained by the first two principal coordinates. Permutational multivariate analysis of variance (Adonis) showed no significant difference in overall microbial community structure between the dietary groups (R^2^ = 0.411, *p* = 0.1).

The 16S rRNA gene was sequenced to evaluate the diversity of the intestinal microbial community in Asian seabass following 56 days of feeding with either control or postbiotic (LSP) diets. Analysis of alpha diversity metrics demonstrated that species richness (Margalef’s d) did not differ significantly between dietary groups. However, the LSP treatment led to reduced community evenness (Pielou’s J’) and lower overall diversity (Shannon and Simpson indices) compared to the control group (Table 4). PCoA based on Bray–Curtis dissimilarity was conducted to evaluate beta diversity. Although some separation was visually apparent in the PCoA plot, Adonis analysis showed that the overall intestinal microbial community structure did not differ significantly between the control and LSP groups (R^2^ = 0.411, *p* = 0.1; Figure 6). The limited biological replication (*n* = 3) and individual variation in gut microbiota may have reduced the statistical power to detect differences in overall community structure.

Taxon-based analysis showed differences in the relative abundance and occurrence of specific bacterial taxa between the control and LSP groups. At the phylum level, eight phyla were detected, while 54 and 67 genera were identified at the genus level in the control and LSP groups, respectively (Figure S1). The abundance of Fusobacteriota increased in the LSP group, whereas Proteobacteria and Firmicutes decreased compared to the control group (Figure S2A,B). Unique genera such as *Salmonella*, *Polaromona*, *Lactococcus*, *Romboutsia*, *Escherichia-Shigella*, and *Staphylococcus* were distinctly identified in the control group, while *Lawsonia*, *Ideonella*, *Methylorubrum*, *Enterococcus*, *Rivibacter*, *Piscinibacter*, *Paucibacter*, and *Fusobacterium* were distinctly identified in the LSP group. *Cetobacterium* was the only genus shared between both dietary treatments (Figure S3). Venn diagrams were constructed to identify core gut taxa present in all seabass samples under different dietary treatments. Seven taxa were shared among all control samples, while 9 taxa were shared among all LSP-fed seabass samples. Comparison of these core microbiome profiles indicated that only 1 core taxon was shared between both dietary groups (Figure S4). Six core taxa were unique to the control group, and 8 core taxa were unique to the LSP group. Collectively, these findings indicate treatment-associated differences in specific microbial taxa between the control and LSP groups, despite the absence of a significant overall shift in gut microbial community structure.

**Figure 7:**
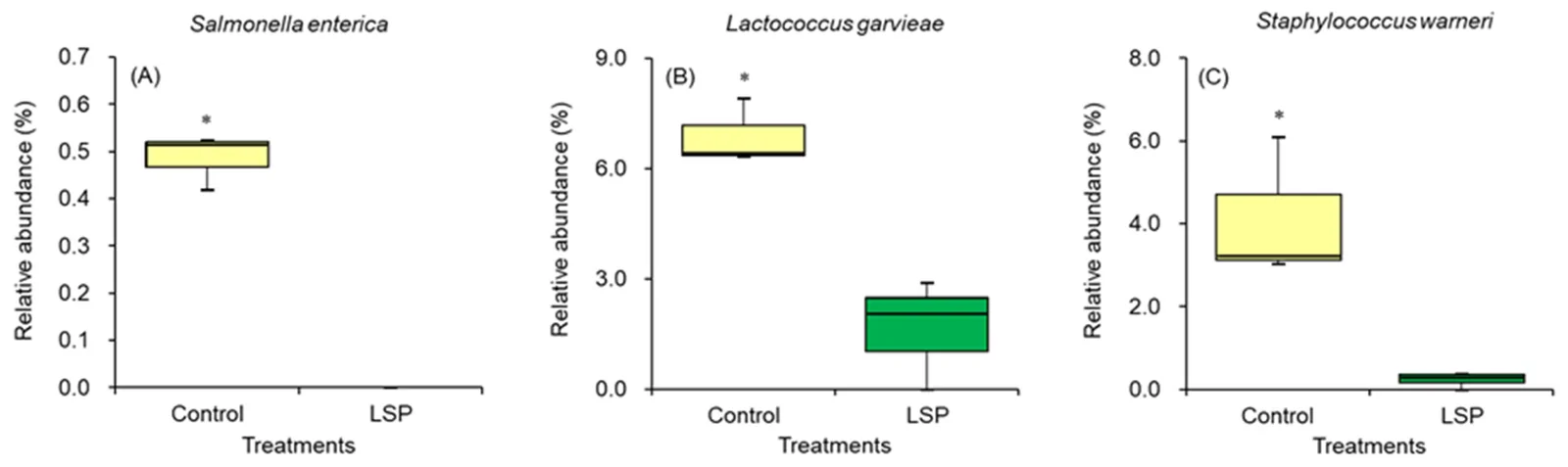
Relative abundance of target pathogenic bacteria across treatments. Comparison of (**A**) *Salmonella enterica*, (**B**) *Lactococcus garvieae*, and (**C**) *Staphylococcus warneri* populations between Control and LSP-supplemented groups. A significant reduction in relative abundance is observed in the LSP group across all three species (* denotes statistical significance).

Significant differences in the relative abundance of selected intestinal bacterial taxa were observed among dietary treatments after 56 days of feeding (Figure 7). The relative abundance of the potential pathogen *Salmonella enterica* (Figure 7A) was significantly lower in the LSP group compared with the control group. LSP treatment significantly reduced the presence of *L. garvieae* compared to the control diet (Figure 7B). Similarly, *Staphylococcus warneri* (Figure 7C) showed a significantly reduced relative abundance in the LSP compared with the control group.

### 3.6. Metabolomics

**Figure 8:**
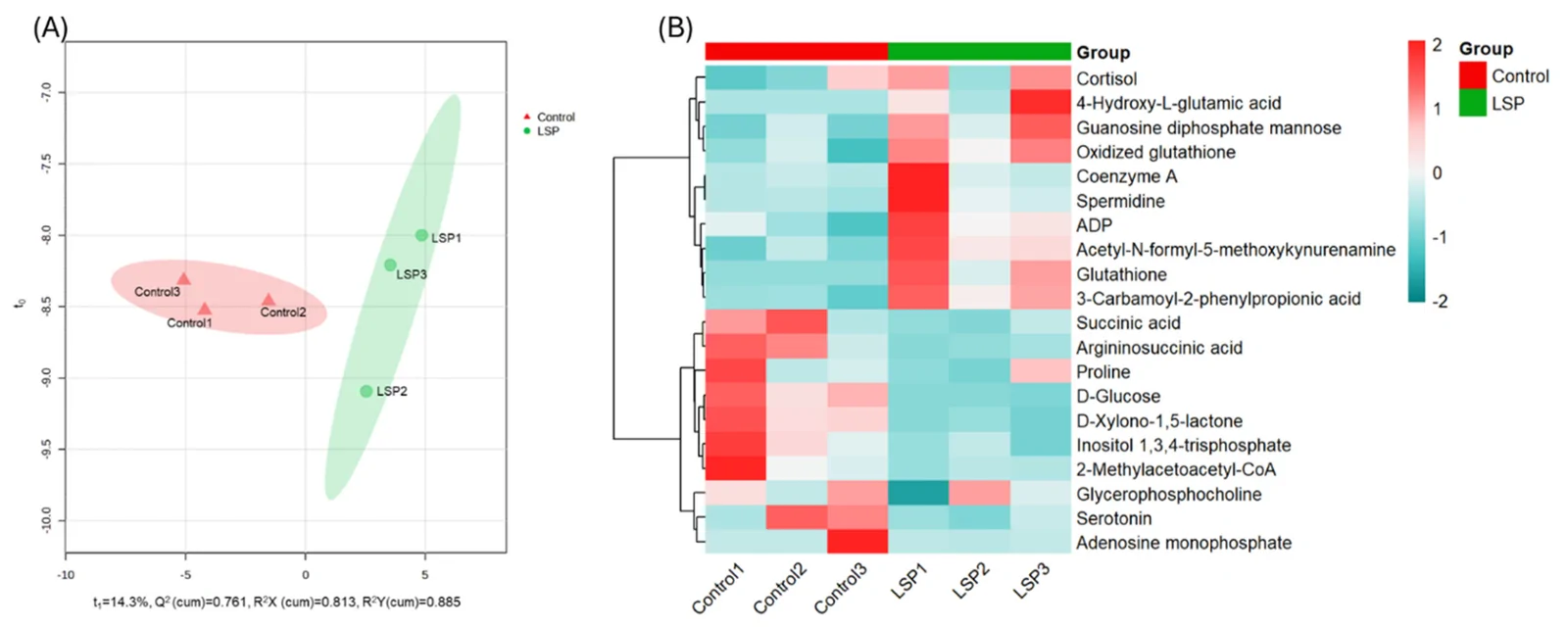
Effects of postbiotic LSP and control treatments on the metabolite profiles of Asian seabass liver tissues. (**A**) OPLS-DA score plot demonstrating metabolic separation between groups. The shaded ellipses indicate the 95% confidence regions for the respective treatment groups. (**B**) Hierarchical clustering heatmap of top 20 differential metabolites, screened based on fold change (FC) and VIP scores > 1.0.

**Figure 9:**
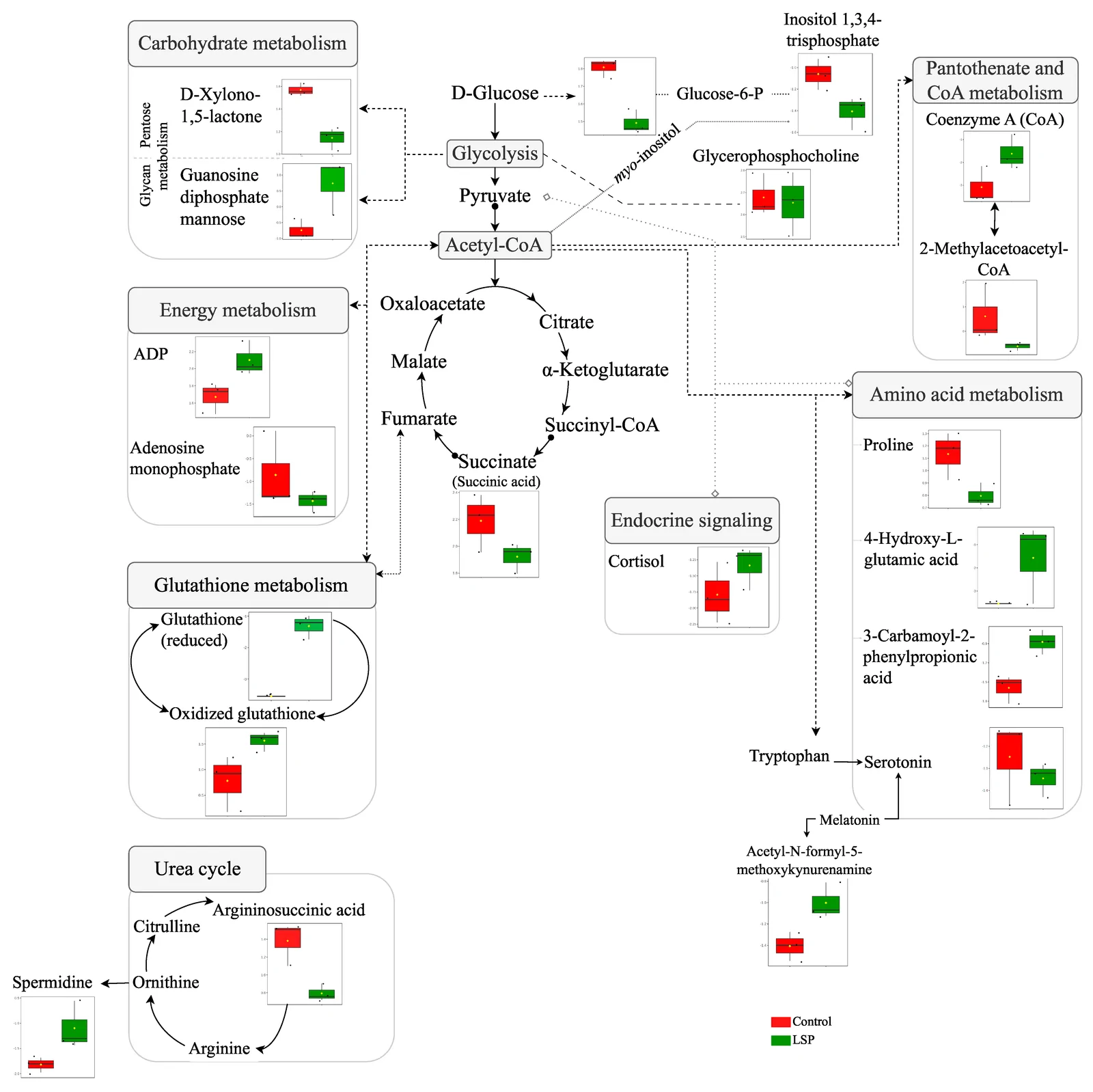
Schematic overview of altered metabolic pathways in liver tissues. The metabolic network is centered on central carbon metabolism, including glycolysis and the tricarboxylic acid (TCA) cycle, with links to peripheral pathways such as amino acid, glutathione, and energy metabolism. The schematic representation is based exclusively on the top 20 metabolites identified through screening with VIP > 1 and fold change thresholds. Box plots illustrate the relative abundance of these metabolites between the Control group (red boxes) and the LSP treatment group (green boxes).

Metabolomic profiling of liver and muscle tissues revealed distinct differences between Asian seabass fed control and postbiotic LSP diets. This distinction was demonstrated by the OPLS-DA plot, which showed complete separation of metabolite profiles between treatment groups, indicating substantial metabolic divergence. The robustness and predictive power of the model were supported by high evaluation metrics (R^2^Y = 0.985; Q^2^ = 0.761), confirming that LSP treatment induced a highly consistent metabolomic shift (Figure 8A). Heatmap analysis of the top 20 discriminant metabolites further indicates these group differences (Figure 8B). The observed metabolomic alterations included changes in carbohydrate, amino acid, and energy metabolism, with significant modulation of the glycolysis pathway, TCA and urea cycles, glutathione metabolism, pantothenate and CoA metabolism, and endocrine signaling. LSP treatment led to increased levels of several metabolites, including guanosine diphosphate mannose, CoA, ADP, reduced and oxidized glutathione, spermidine, 4-hydroxy-L-glutamic acid, 3-carbamoyl-2-phenylpropionic acid, acetyl-N-formyl-5-methoxykynurenamine, and cortisol. In contrast, D-xylono-1,5-lactone, D-glucose, glucose-6-phosphate, inositol 1,3,4-trisphosphate, 2-methylacetoacetyl-CoA, AMP, succinate, argininosuccinic acid, proline, and serotonin were elevated in the control group (Figure 9).

**Figure 10:**
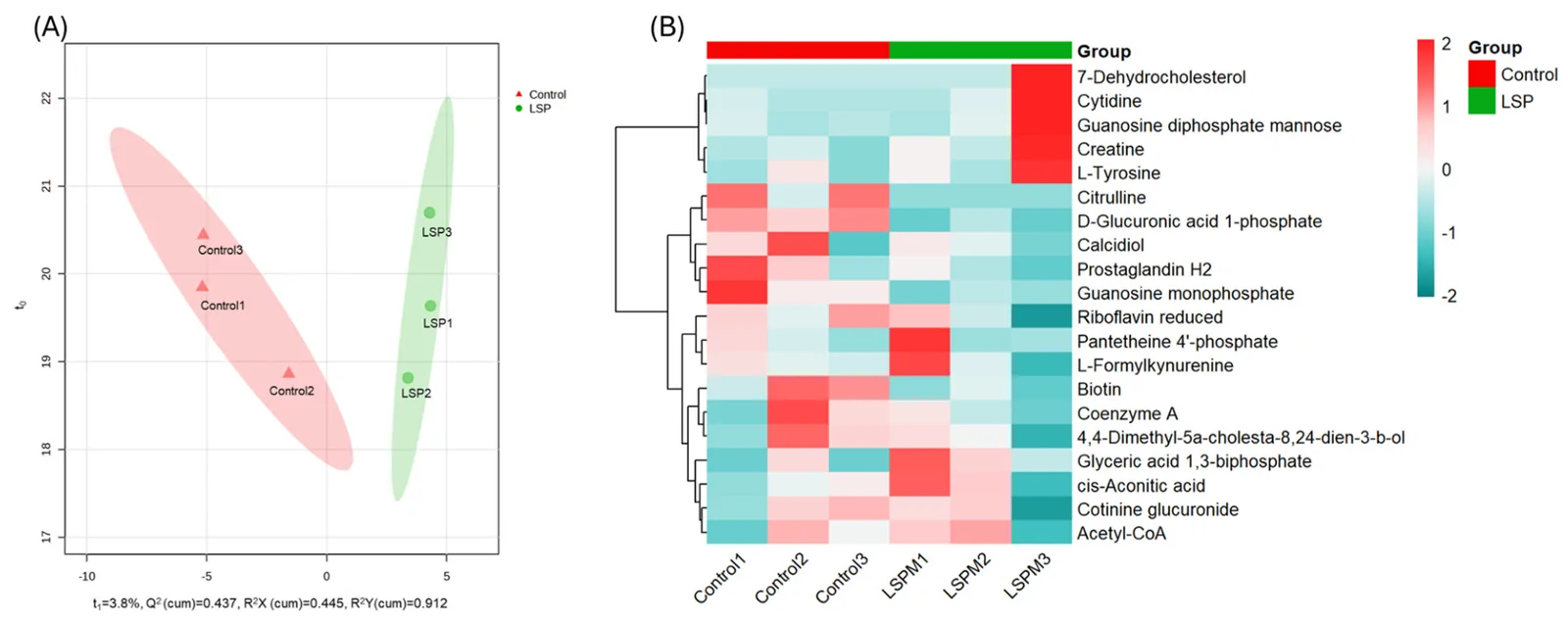
Effects of postbiotic LSP and control treatments on the metabolite profiles of Asian seabass muscle tissues. (**A**) OPLS-DA score plot demonstrating metabolic separation between groups. The shaded ellipses indicate the 95% confidence regions for the respective treatment groups. (**B**) Hierarchical clustering heatmap of top 20 differential metabolites, screened based on fold change (FC) and VIP scores > 1.0.

**Figure 11:**
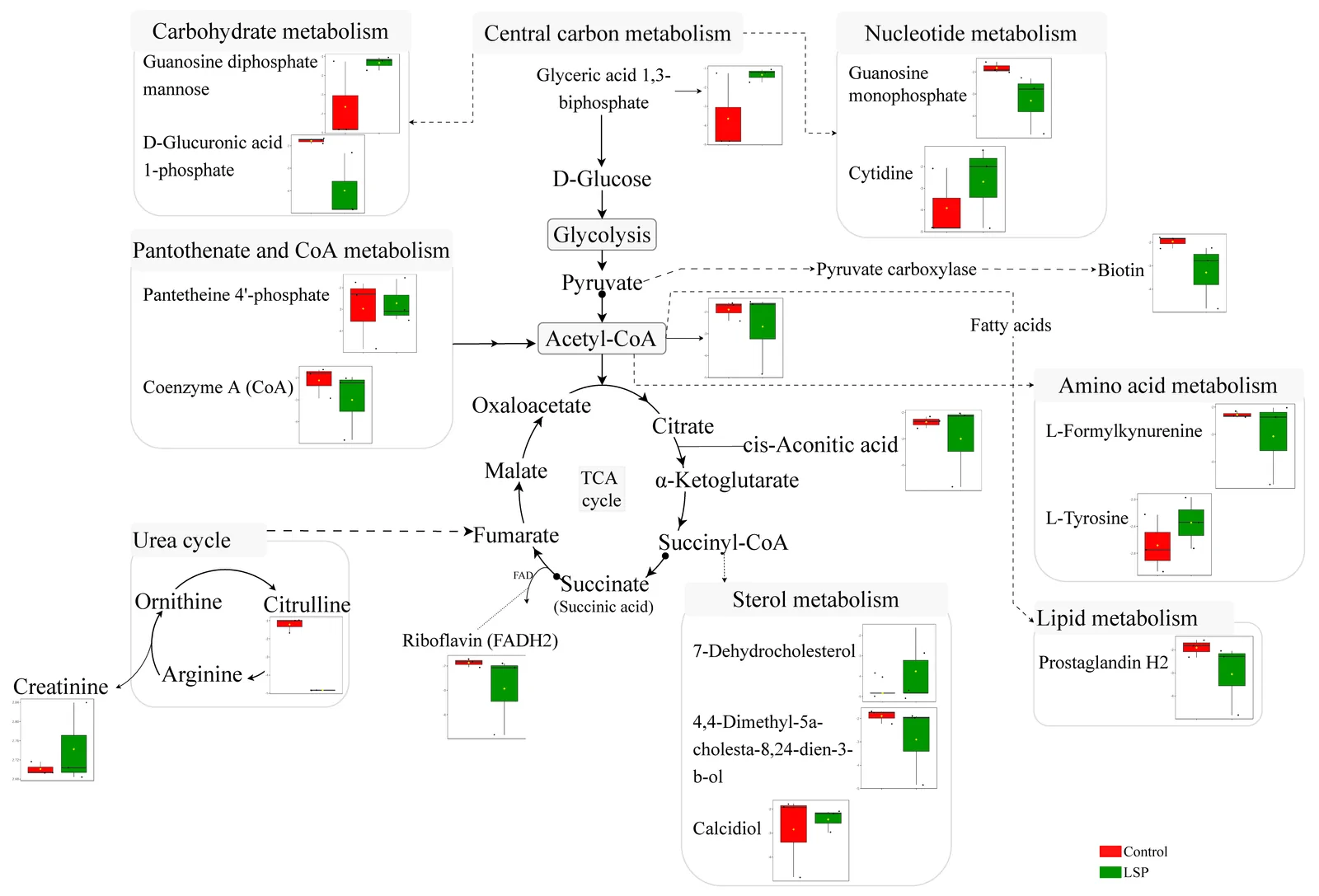
Schematic overview of altered metabolic pathways in muscle tissues. The metabolic network is centered around central carbon metabolism, including glycolysis and the tricarboxylic acid (TCA) cycle, with links to pathways including carbohydrate, nucleotide, lipid, sterol, amino acid, and pantothenate/CoA metabolism, alongside the urea cycle. The schematic representation is based exclusively on the top 20 metabolites identified through screening with VIP > 1 and fold change thresholds. Box plots show the relative abundance of altered metabolites between the Control group (red boxes) and the LSP treatment group (green boxes).

Similarly, the muscle tissue revealed that postbiotic LSP induced distinct shifts in the metabolite profile compared to control diets. OPLS-DA modeling demonstrated clear metabolic separation between groups (R^2^Y = 0.985 and Q^2^ = 0.761) (Figure 10A). The heatmap showed the relative abundance of the top 20 metabolites that differed among treatments in muscle (Figure 10B). Metabolic changes included regulations in carbohydrate, amino acid, nucleotide, and lipid metabolism, with significant modulation of central metabolic pathways. Increased levels of several including metabolites involved in energy and antioxidant pathways were observed in the LSP treatment, while control diets maintained higher abundances of metabolites linked to intermediary metabolism (Figure 11).

**Figure 12:**
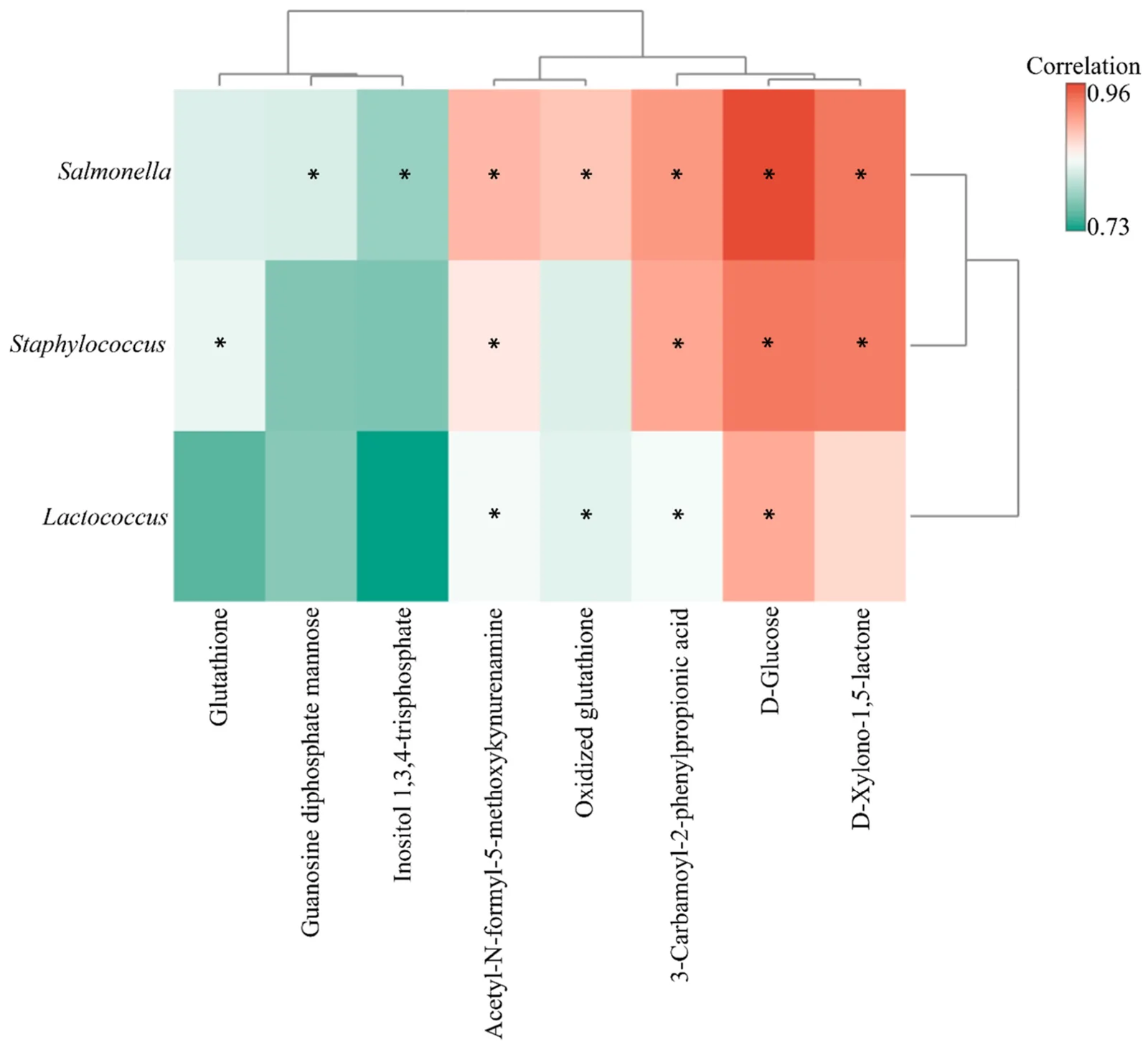
Correlation heatmap between targeted metabolites and microbial genera. Hierarchical clustering heatmap displaying Spearman’s rank correlation coefficients between key bacterial taxa (y-axis) and significantly altered metabolites (x-axis). The color gradient represents the strength of the correlation. Asterisks (*) denote statistically significant correlations (*p* < 0.05). Top and right dendrograms show the hierarchical clustering of the metabolites and microbial genera, respectively, based on the similarity of their correlation profiles.

Correlation networks between the metabolome and potentially pathogenic taxa, including *Salmonella*, *Staphylococcus*, and *Lactococcus*, were identified by integrative analysis using Spearman’s rank correlation analysis. Significant positive correlations (*p* < 0.05) were observed between the relative abundance of these taxa and energy- or stress-associated metabolites, including D-glucose, 3-carbamoyl-2-phenylpropionic acid, D-xylono-1,5-lactone, and oxidized glutathione. In contrast, strong negative associations were observed with guanosine diphosphate mannose, inositol 1,3,4-trisphosphate, and reduced glutathione (Figure 12).

## 4. Discussion

### 4.1. Growth and Survival Performance

Postbiotics have been increasingly used as dietary additives in aquaculture because they may improve growth and feed utilization through enhanced nutrient availability, digestive activity, intestinal function, and gut microbial balance [28]. For example, *L*. *plantarum* postbiotics improved growth and feed utilization in juvenile black sea bream [29], while heat-killed *L. plantarum* L-137 enhanced growth, feed efficiency, and survival in bighead catfish [30]. However, these effects vary with fish species, postbiotic source, dosage, and culture conditions. Heat-killed *L. plantarum* and *Bacillus* sp. SJ-10, for instance, did not significantly affect the growth of juvenile olive flounder [31]. Similarly, SYNSEA Premium supplementation did not significantly improve the growth performance of Asian seabass in the present study. Although postbiotic supplementation did not significantly affect growth performance or protein utilization, survival was significantly higher in the postbiotic-fed groups than in the control group. This improvement may be relevant under commercial conditions, where increased survival can contribute to greater biomass production. Because cannibalism is an important cause of mortality in juvenile Asian seabass, particularly when size variation is high [32,33], the survival of smaller fish may have reduced the mean body weight and partly masked the growth-promoting effect. Postbiotic supplementation did not significantly affect dorsal muscle proximate composition or fatty acid profiles (Table 3 and Table S2). Therefore, although changes in muscle metabolic profiles were observed, these metabolic alterations were not accompanied by detectable changes in muscle nutrient composition under the present experimental conditions.

### 4.2. Disease Resistance and Innate Immunity

Postbiotics have been increasingly investigated as biocontrol agents for disease management in aquaculture [34]. Heat-inactivated probiotics from several bacterial genera can improve host health, suggesting that microbial cell components and metabolites may modulate immune responses even in the absence of viable cells [35]. For example, heat-killed *L*. *paracasei* reduced *V*. *harveyi* loads in Japanese pufferfish after challenge [36], while heat-killed *Weissella cibaria* enhanced the resistance of rainbow trout to *Yersinia ruckeri* and regulated the expression of immune-related cytokines [37]. Although probiotics can improve antiviral resistance in fish [38], evidence regarding the antiviral effects of postbiotics remains limited. Studies in mammalian models have shown that heat-killed *L. plantarum* L-137 can stimulate Th1-associated cytokines, including IL-12 and IFN-γ, and enhance protection against influenza virus infection [39,40,41]. In the present study, SYNSEA Premium supplementation increased the resistance of Asian seabass to both *V. alginolyticus* and iridovirus, suggesting that its health benefits were not restricted to a single pathogen type.

Phagocyte-associated responses, including respiratory burst activity, antioxidant defense, and phagocytic capacity, are important indicators of innate immune function in fish. During phagocytosis, activation of NADPH oxidase generates ROS, particularly superoxide anions, which contribute to the destruction of invading microorganisms. However, excessive ROS production may cause oxidative damage to host cells. SOD therefore plays an important protective role by converting superoxide anions into hydrogen peroxide and molecular oxygen, thereby helping to maintain redox balance during immune activation [42]. Previous studies have shown that dietary postbiotics or heat-inactivated probiotics can enhance cellular immune and antioxidant responses in fish. Heat-inactivated *B*. *subtilis* increased lysozyme, phagocytic, and antioxidant enzyme activities in striped catfish [43], whereas heat-killed *L*. *plantarum* VSG3 enhanced phagocytic activity, complement activity, and antioxidant defenses in common carp [44]. In the present study, SYNSEA Premium significantly increased SOD and phagocytic activities without significantly affecting respiratory burst activity. These findings indicate enhanced cellular immune and antioxidant responses following postbiotic supplementation.

Lysozyme is an important humoral defense enzyme in fish innate immunity and contributes to the elimination of pathogenic microorganisms through bacteriolytic activity [45]. By hydrolyzing peptidoglycan in bacterial cell walls, lysozyme can directly damage bacteria and may also act synergistically with other immune mechanisms, including complement activity, phagocytosis, and oxidative killing. Several studies have shown that dietary postbiotics or heat-inactivated probiotics enhance lysozyme activity in aquatic species, such as striped catfish fed heat-inactivated *B. subtilis* [43], common carp fed heat-killed *L. plantarum* VSG3 [44], and bighead catfish fed heat-killed *L. plantarum* L-137 [30]. Consistent with these studies, lysozyme activity was significantly increased in postbiotic-fed Asian seabass. The concurrent enhancement of phagocytic activity, SOD activity, and lysozyme activity provides physiological evidence that SYNSEA Premium enhanced several components of innate immune competence, which may be associated with the greater disease resistance observed in the challenge experiments.

Dietary SYNSEA Premium did not broadly activate all immune-related genes under basal conditions but selectively affected several immune regulators, including *ifn-γ1*, *tgf-β1*, *tnf*, and *c3*. IFN-γ is involved in antimicrobial and antiviral immune responses in teleosts [46,47], whereas TNF participates in inflammatory signaling and leukocyte activation [48]. TGF-β1 has important regulatory functions in inflammation and immune homeostasis [49,50], and C3 is a central component of the complement system involved in opsonization and phagocytic clearance [51,52]. The observed changes in these genes are therefore consistent with modulation of innate immune responses following postbiotic supplementation. However, because C3 protein concentrations and complement activity were not directly measured, the functional consequences of changes in *c3* expression should be interpreted cautiously.

Following NNV challenge, increased splenic *mx* expression was observed in postbiotic-fed fish. Mx proteins are interferon-inducible antiviral effectors and are commonly used as markers of antiviral immune activation in fish [53]. However, only *mx* was examined after viral stimulation in the present study. Upstream components of the retinoic acid-inducible gene I-like receptor (RLR) signaling pathway, including *rig-i*, *mavs*, *irf3*, and *irf7*, were not evaluated. Therefore, the present results indicate modulation of an antiviral marker but do not demonstrate activation of the RLR pathway or establish the molecular mechanism underlying the improved resistance to viral infection. Further studies examining upstream antiviral signaling will be required to clarify this mechanism.

### 4.3. Gut Microbiota Composition

The overall intestinal microbial community structure did not differ significantly between the control and LSP groups according to the Adonis analysis, although differences in the relative abundance of several bacterial taxa were observed. Fusobacteriota remained a dominant phylum in both groups and was relatively more abundant in the LSP group, whereas Proteobacteria was more abundant in the control group. Similar enrichment of Fusobacteriota has been reported in the intestinal microbiota of carnivorous marine fish and other fish species [54,55,56,57]. At the taxon level, several potentially pathogenic or opportunistic bacteria, including *Salmonella*, *Escherichia–Shigella*, *Lactococcus*, and *Staphylococcus*, were more abundant in the control group. These findings therefore indicate treatment-associated differences in specific bacterial taxa rather than a global restructuring of the intestinal microbial community.

Among the dominant intestinal taxa, *Cetobacterium* was more abundant in LSP-fed fish. This genus is widely distributed in fish intestines and has previously been associated with acetate production, glucose metabolism, vitamin B12 synthesis, intestinal function, and resistance to infection [58,59,60,61]. Its higher relative abundance in the LSP group represents a treatment-associated taxon-level difference; however, its functional contribution was not directly evaluated in the present study. Members of Fusobacteriota, particularly *Cetobacterium*, have also been associated with short-chain fatty acid production and carbohydrate and amino acid metabolism [62,63,64]. In parallel, hepatic riboflavin and reduced riboflavin levels were elevated in fish fed the LSP diet (Figure S5). The concurrent changes in specific intestinal taxa and hepatic riboflavin-related metabolites suggest a potential association between gut microbial composition and host metabolic responses; however, microbial metabolic activity was not directly measured, and no causal or functional relationship can be established from the present data. A major limitation of the present study is that mucosal immune responses and intestinal histology were not evaluated. Therefore, the observed changes in intestinal microbial taxa cannot be interpreted as direct evidence of improved mucosal immunity or intestinal epithelial integrity. Further studies incorporating mucus-associated immune parameters and histological assessment of the intestine will be needed to determine whether the microbiota-associated changes are accompanied by improvements in intestinal health.

### 4.4. Metabolic Responses and Microbiome-Metabolome Associations

SYNSEA Premium consisted of inactivated bacterial cells without additional defined bioactive metabolites [8,65,66]. Therefore, the metabolomic changes observed in the fish were interpreted as host metabolic responses associated with dietary postbiotic supplementation rather than as direct contributions of metabolites contained in the product.

Several metabolites altered by SYNSEA Premium supplementation were related to central carbon and CoA-dependent metabolism. CoA is an essential metabolic cofactor linking carbohydrate, amino acid, and lipid metabolism, while acetyl-CoA serves as a major intermediate connecting these pathways with the tricarboxylic acid cycle and biosynthetic metabolism [67,68]. The increased CoA level together with the decreased 2-methylacetoacetyl-CoA level was consistent with differences in CoA-related metabolic profiles. Because 2-methylacetoacetyl-CoA is associated with branched-chain amino acid degradation, particularly isoleucine catabolism, these changes may reflect differences in amino acid- and CoA-related metabolism. Nevertheless, metabolite abundance does not directly represent metabolic flux, and the present data therefore indicate changes in metabolic profiles rather than increased or decreased pathway activity.

Postbiotic supplementation was also associated with changes in glutathione metabolism. Reduced glutathione (GSH) is a major non-enzymatic antioxidant that contributes to cellular redox regulation and protection against reactive oxygen species. Previous studies have shown that nutritional interventions, including probiotics and prebiotics, can influence the glutathione antioxidant system [69], and dietary GSH supplementation has been reported to improve antioxidant capacity and intestinal physiological status in *Oncorhynchus mykiss* [70]. The higher GSH level in LSP-fed fish occurred together with increased SOD activity, indicating concurrent changes in a non-enzymatic antioxidant metabolite and an antioxidant enzyme. However, oxidative damage and metabolic flux were not measured, and the functional relationship between these responses remains uncertain.

The integrated microbiome–metabolome analysis further identified biologically relevant associations between specific intestinal bacterial taxa and host metabolites. The relative abundances of potentially pathogenic taxa, including *Salmonella*, *Staphylococcus*, and *Lactococcus*, were positively associated with D-glucose. Glucose is an important carbon and energy source for both host cells and microorganisms, and previous studies have shown that *Salmonella* can exploit host glucose metabolism during intracellular infection, including through regulation of host glucose uptake pathways [71]. The lower relative abundance of *Salmonella* in LSP-fed fish coincided with lower hepatic D-glucose and D-xylono-1,5-lactone levels. These concurrent changes suggest an association between intestinal bacterial composition and host carbohydrate-related metabolites; however, the correlation analysis does not establish the direction or causality of this relationship.

Associations involving glutathione-related metabolites were also observed. *Lactococcus* abundance was positively associated with oxidized glutathione, whereas *Staphylococcus* showed a negative association with reduced glutathione. Together with the lower D-glucose and D-xylono-1,5-lactone levels and higher reduced glutathione level observed in LSP-fed fish, these findings indicate potential relationships among intestinal bacterial composition, host carbohydrate metabolism, and redox status. These correlations are biologically plausible but should be regarded as exploratory and hypothesis-generating rather than evidence that postbiotic-induced metabolic changes directly suppressed bacterial colonization. No functional experiments were performed to validate these associations, and the limited biological replication of the omics analyses further restricts mechanistic interpretation.

An additional limitation is that the microbiome and metabolome analyses were performed only in the control and LSP groups, because corresponding omics data were not available for the HSP group. Consequently, the present study cannot determine whether the microbial and metabolic changes observed in the LSP group also occur at the higher supplementation level or follow a dose-dependent pattern. The omics findings should therefore be interpreted specifically as responses associated with LSP supplementation rather than as evidence of a dose-dependent effect of SYNSEA Premium. Future studies including both supplementation levels, together with greater biological replication and functional validation, will be needed to determine whether these responses vary with supplementation level.

## 5. Conclusions

Dietary supplementation with SYNSEA Premium did not significantly affect growth performance or dorsal muscle composition in Asian seabass but significantly improved survival, innate immune responses, and resistance to bacterial and viral infections. Exploratory omics analyses conducted only in the control and LSP groups identified differences in the relative abundance of specific intestinal bacterial taxa and changes in host metabolic profiles. These omics findings are specific to the LSP comparison and cannot be used to infer dose-dependent microbial or metabolic responses. Integrated microbiome–metabolome analysis identified correlations between specific microbial taxa and host metabolites; however, these associations should be considered exploratory and do not establish causal or mechanistic relationships. Overall, the results support the potential use of SYNSEA Premium as a functional feed additive for improving health and disease resistance in Asian seabass.

## Figures and Tables

**Table 1:** Experimental diet ingredients used in this study. Proximate composition data are expressed as mean ± S.E.

Ingredients	Experimental Diets (g kg^−1^)		
	Control	LSP	HSP
Fish meal	532	532	532
Dehulled soybean meal	200	200	200
Gluten	20	20	20
α-Starch	90	90	90
Fish oil	35	35	35
Shrimp meal	60	60	60
Min. premix ^a^	20	20	20
Vit. premix ^b^	15	15	15
HSP postbiotic	0	0	10
LSP postbiotic	0	10	0
α-Cellulose	28	18	18
Proximate analysis of the diets			
Crude protein (%)	50.26 ± 0.43	50.71 ± 0.18	50.99 ± 0.3
Ether extract (%)	8.24 ± 0.59	8.39 ± 0.82	8.9 ± 1.56
Ash (%)	15.32 ± 0.1	15.35 ± 0.73	15.85 ± 0.23
Moisture (%)	2.93 ± 0.06	2.75 ± 0.31	2.98 ± 0.11

^a^ Mineral premix (provided by Nice Garden Industrial Co., Ltd., Taipei, Taiwan; per kg premix): Ca, 140.00 g; P, 150.00 g; Mg, 18.50 g; K, 35.50 g; Cu, 0.45 g; I, 0.01 g; Fe, 5.05 g; Mn, 1.03 g; Se, 0.01 g; Zn, 2.80 g; and Co, 0.03 g. ^b^ Vitamin premix (provided by Nice Garden Industrial Co., Ltd., Taipei, Taiwan; per kg premix): vitamin A, 12,500,000 IU; vitamin D_3_, 2,500,000 IU; vitamin E, 111,000 IU; vitamin K_3_, 14.25 g; vitamin B_1_, 16.10 g; vitamin B_2_, 13.90 g; vitamin B_6_, 13.25 g; vitamin B_12_, 100 mg; niacin, 66.50 g; calcium pantothenate, 34.40 g; folic acid, 4.31 g; biotin, 650 mg; vitamin C, 45.00 g; and inositol, 75.00 g.

**Table 2:** Growth performance of fish after 56 days of feeding experimental diets. Data are mean ± SE (*n* = 3), where n represents the number of tanks. Different superscript letters within the same row indicate significant differences among treatments (*p* < 0.05); values with different letters differ significantly (*p* < 0.05).

Parameters	Experimental Diets		
	Control	LSP	HSP
Final weight (g)	58.8 ± 1.6	58.7 ± 1.0	58.3 ± 1.7
Survival (%)	90.0 ± 1.4 ^b^	97.3 ± 0.8 ^a^	95.3 ± 0.8 ^a^
Percentage of weight gain (%)	902.4 ± 37.7	901.7 ± 24.3	894.6 ± 40.6
Feed efficiency	1.16 ± 0.02	1.21 ± 0.01	1.21 ± 0.02
Protein efficiency ratio	2.31 ± 0.06	2.38 ± 0.01	2.37 ± 0.04
Total production (g) per tank	2642.6 ± 31.6	2858.1 ± 72.6	2779.2 ± 88.0
Condition factor	1.94 ± 0.10	2.06 ± 0.07	2.05 ± 0.04

**Table 3:** Proximate composition of the dorsal muscle of Asian seabass fed a control diet without postbiotics or diets supplemented with low-dose postbiotics (LSP; 1 × 10^8^ cells kg^−1^ diet) or high-dose postbiotics (HSP; 1 × 10^9^ cells kg^−1^ diet) for 56 days. Values are expressed as the mean ± SE (*n* = 3 replicate cages).

Parameters	Experimental Diets		
	Control	LSP	HSP
Moisture (%)	74.18 ± 0.3	73.94 ± 0.72	74.37 ± 0.41
Crude protein (% in dry weight)	88.44 ± 0.65	87.21 ± 0.60	87.55 ± 0.43
Crude lipid (% in dry weight)	4.30 ± 0.79	4.56 ± 0.51	4.95 ± 0.48
Ash (% in dry weight)	5.85 ± 0.15	5.65 ± 0.08	5.97 ± 0.13

**Table 4:** Microflora diversity and richness indices relative to dietary treatments. Values represent mean ± SE (*n* = 3), where fish were sampled evenly from different tanks.

Treatments	Genus	Margalef’s Species Richness (d)	Pielou’s Evenness (J’)	Shannon Index	Simpson Index
Control	54	3.49 ± 0.40	0.52 ± 0.11	1.79 ± 0.43	0.62 ± 0.14
LSP	67	3.57 ± 0.87	0.37 ± 0.08	1.31 ± 0.37	0.43 ± 0.10

## Data Availability

The original contributions presented in this study are included in the article/Supplementary Material. Further inquiries can be directed to the corresponding author.

## Supplementary Materials

The following supporting information can be downloaded at https://www.mdpi.com/article/10.3390/biology15181605/s1, Figure S1: Numbers of intestinal microbial taxa detected at the phylum, class, order, family, and genus levels in Asian seabass fed a control diet without postbiotics or a low-dose postbiotic diet (LSP; 1 × 10^8^ cells kg^-1^ diet) for 56 days. The genus-level comparison shows the total number of genera and the numbers detected in the control and LSP groups. Figure S2: Relative abundance of dominant bacterial phyla in the intestinal microbiota of Asian seabass fed a control diet without postbiotics (A) or a low-dose postbiotic diet (LSP; 1 × 10^8^ cells kg^-1^ diet) (B) for 56 days. The relative abundance of each bacterial phylum is expressed as a percentage of the total bacterial sequences obtained within each treatment group Figure S3: Relative abundance of major bacterial genera in the intestinal microbiota of Asian seabass fed a control diet without postbiotics or a low-dose postbiotic diet (LSP; 1 × 10^8^ cells kg^-1^ diet) for 56 days. Relative abundance is expressed as the percentage of total bacterial sequences detected in each treatment group. Figure S4: Venn diagram showing the shared and unique intestinal microbial genera detected in Asian seabass fed a control diet without postbiotics (A) or a low-dose postbiotic diet (LSP; 1 × 10^8^ cells kg^-1^ diet) (B) for 56 days. The overlapping area indicates genera common to both treatment groups, whereas the non-overlapping areas indicate genera unique to each group (C). Numbers represent the total genera identified in each category. Figure S5: Relative abundances of riboflavin (A) and reduced riboflavin (B) in the liver of Asian seabass fed a control diet without postbiotics or a low-dose postbiotic diet (LSP; 1 × 10^8^ cells kg^-1^ diet) for 56 days. Neither metabolite met the variable importance in projection (VIP) screening criterion of VIP > 1. The fold change (FC) and p value were 1.35 and 0.252 for riboflavin and 2.44 and 0.0186 for reduced riboflavin, respectively. Table S1: Primers used for q-PCR. Table S2: Fatty acid composition of the dorsal muscle of Asian seabass fed a control diet without postbiotics or diets supplemented with low-dose postbiotics (LSP; 1 × 10^8^ cells kg^-1^ diet) or high-dose postbiotics (HSP; 1 × 10^9^ cells kg^-1^ diet) for 56 days. Fatty acid contents are expressed as percentages of total identified fatty acids. Values are presented as the mean ± SE (*n* = 3 replicate cages).
