# Supplementary material for: Integrative Analysis of Gut Microbiota and Metabolome Reveals Health Benefits of Postbiotic Supplementation in Asian Seabass (Lates calcarifer)
Source: Biology (Basel). 2026 Sep 11;15(18):1605. doi: 10.3390/biology15181605 (PMC13604681; doi:10.3390/biology15181605)
Supplement: Supplementary file 1 [file biology-15-01605-s001.zip › biology-4517154-supplementary.pdf]

Table S1. Primers used for q-PCR

Genes	Primer sequence (5'→3')	References
crp	F: ACCGAACTGAAGACCACGAT<br>R: TGGGGCACCTCAAACAAA	
c3	F: AAATGCTGCCATCGTTCC<br>R: CCAGTGACCTTCAGACCAAA	
c4	F: CGAGGTTGAACGAAAAGGAC<br>R: CACAGCAAGCAAAGCCACT	
mtor	F: GTTTCTTCCGCTCCATTTC<br>R: CAGGGCTTCATTCACTTCA	
mlst-8	F: TGATTCAACACTATTAGCCACA<br>R: TTTCCACGCACCACAGG	
eif4e	F: TGACGACTACAGCGATGAT<br>R: GTGTCTGCGTGGGATTG	
hsp70	F: CTGGAGTCCTACGCTTTCAA<br>R: CTTGCTGATGATGGGGTTAC	
hsp90	F: ACGATGATGAGCAGTATGCC<br>R: CAAACAGGGTGATGGGGTA	
il-8	F: TCTGACTGTTCCTGAGGCTATC<br>R: GACGTCCAATGGGCTTTCT	
il-10	F: TGCTGCCGTTTTGTGGAG<br>R: ACCGTGCTCAGGTAAAAGTCC	
tgf-β1	F: TACCTCGCTTCCCGTTTC<br>R: CTGCTCATCCTCAGTCCCTC	
tnf	F: AAGGACTCCGCTGAGAAAAC<br>R: TGAACGATGCCTGGCTGTA	
ifn-γ1	F: TACCAGGAGCAGGACAAGC<br>R: TCGTCAGGCAGCGAACTT	
mx	F: TGAGGAGAAGGTGCGTCC<br>R: GCGCCTCCAACACGGAGCTC	
β-actin	F: CACTCAACCCCAAAGCCAACAGG<br>R: AAAGTCCAGCGCCACGTAGCACAG	

Note: C-reactive protein (*crp*), Complement C-3 (*c3*), Complement C-4 (*c4*), Mechanistic target of rapamycin (*mtor*), Mammalian lethal with SEC13 protein 8 (*mlst-8*), Eukaryotic translation initiation factor 4E (*eif4e*), Heat shock cognate 70 kDa protein (*hsp70*), Heat shock cognate 90 kDa protein (*hsp90*), Interleukin-8 (*il-8*), Interleukin-10 (*il-10*), Transforming growth factor β-1 (*tgf-β1*), Tumor necrosis factor (*tnf*), Interferon γ1 (*ifn-γ1*), and myxovirus resistance protein (*mx*).

Table S2. Fatty acid composition of the dorsal muscle of Asian seabass fed a control diet without postbiotics or diets supplemented with low-dose postbiotics (LSP; 1×10^8 cells kg^{-1} diet) or high-dose postbiotics (HSP; 1×10^9 cells kg^{-1} diet) for 56 days. Fatty acid contents are expressed as percentages of total identified fatty acids. Values are presented as the mean ± SE (n = 3 replicate cages)

Fatty acids (% of total fatty acids)	Control	LSP	HSP
C14:0	3.51±0.33	3.15±1.13	3.39±1.23
C16:0	24.16±1.76	23.91±0.44	24.83±1.84
C18:0	6.49±0.84	6.67±0.42	6.74±0.48
C16:1	8.10±0.71	7.76±0.32	7.69±1.05
C18:1n9t	1.60±0.68	1.12±0.09	1.33±0.17
C18:1n9c	19.07±4.56	21.52±0.62	19.23±4.99
C20:1	2.59±0.20	2.31±0.14	2.74±0.24
C22:1n9	3.14±0.23	2.75±0.23	3.24±0.37
C18:2n6t	3.99±1.08	4.35±0.29	3.78±1.33
C18:2n6c	5.91±0.63	6.04±0.52	5.87±0.97
C18:3n6	3.89±0.29	3.47±0.19	4.23±0.35
C20:4n6	0.80±0.15	0.75±0.15	0.77±0.13
C20:2(C20:2n6)	0.95±0.09	0.95±0.11	0.91±0.14
C22:2(C22:2n6)	0.36±0.03	0.28±0.06	0.20±0.13
C18:3n3	0.68±0.10	0.65±0.12	0.56±0.22
C20:5n3	6.63±0.98	6.90±0.48	6.91±0.70
C22:6n3	8.12±0.86	7.43±0.93	7.60±0.71
Saturated	34.16±2.14	33.73±1.04	34.96±2.96
MUFA	34.50±2.98	35.47±1.03	34.23±3.96
PUFA	31.33±1.24	30.81±0.93	30.81±1.72
(n-3)PUFA	15.43±1.54	14.98±0.65	15.06±1.51
(n-3)HUFA	14.75±1.57	14.33±0.63	14.51±1.36
n-6	15.91±0.77	15.83±0.53	15.75±0.41
n-3/n-6	0.98±0.13	0.95±0.05	0.96±0.09
DHA/EPA	1.26±0.20	1.10±0.18	1.10±0.06

Abbreviations: LSP, low-dose postbiotic supplementation; HSP, high-dose postbiotic supplementation; SFA, saturated fatty acids; MUFA, monounsaturated fatty acids; PUFA, polyunsaturated fatty acids; HUFA, highly unsaturated fatty acids; EPA, eicosapentaenoic acid; DHA, docosahexaenoic acid; c, cis; t, trans; SE, standard error.

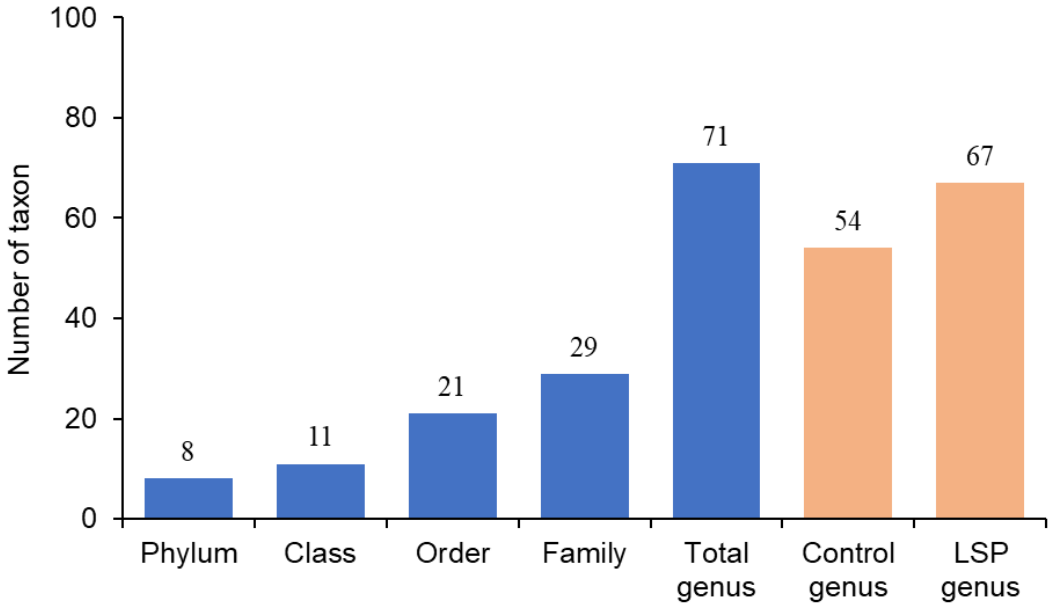

Figure S1. Numbers of intestinal microbial taxa detected at the phylum, class, order, family, and genus levels in Asian seabass fed a control diet without postbiotics or a low-dose postbiotic diet (LSP; 1×10^8 cells kg^{-1} diet) for 56 days. The genus-level comparison shows the total number of genera and the numbers detected in the control and LSP groups.

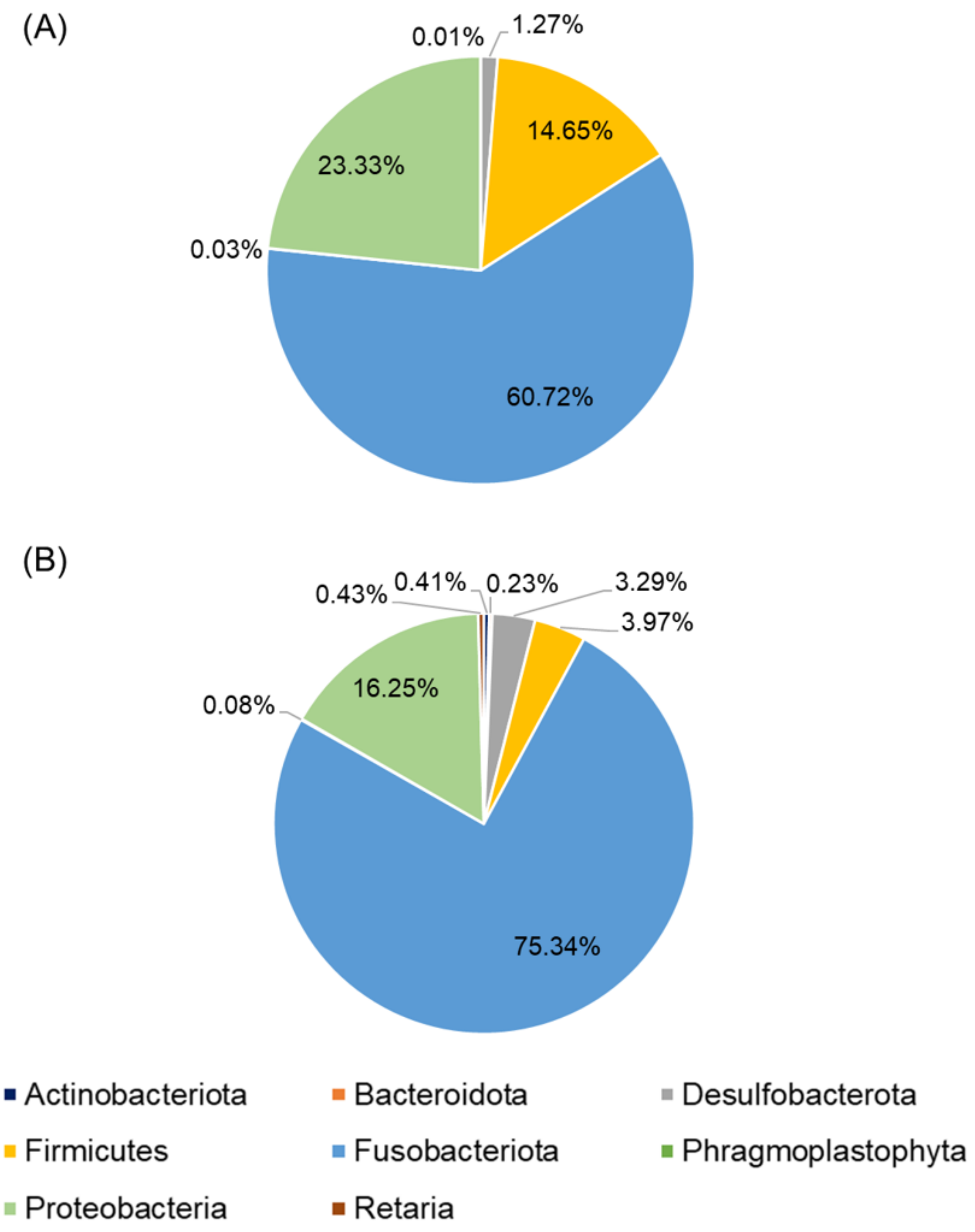

Figure S2. Relative abundance of dominant bacterial phyla in the intestinal microbiota of Asian seabass fed a control diet without postbiotics (A) or a low-dose postbiotic diet (LSP; 1×10^8 cells kg^{-1} diet) (B) for 56 days. The relative abundance of each bacterial phylum is expressed as a percentage of the total bacterial sequences obtained within each treatment group.

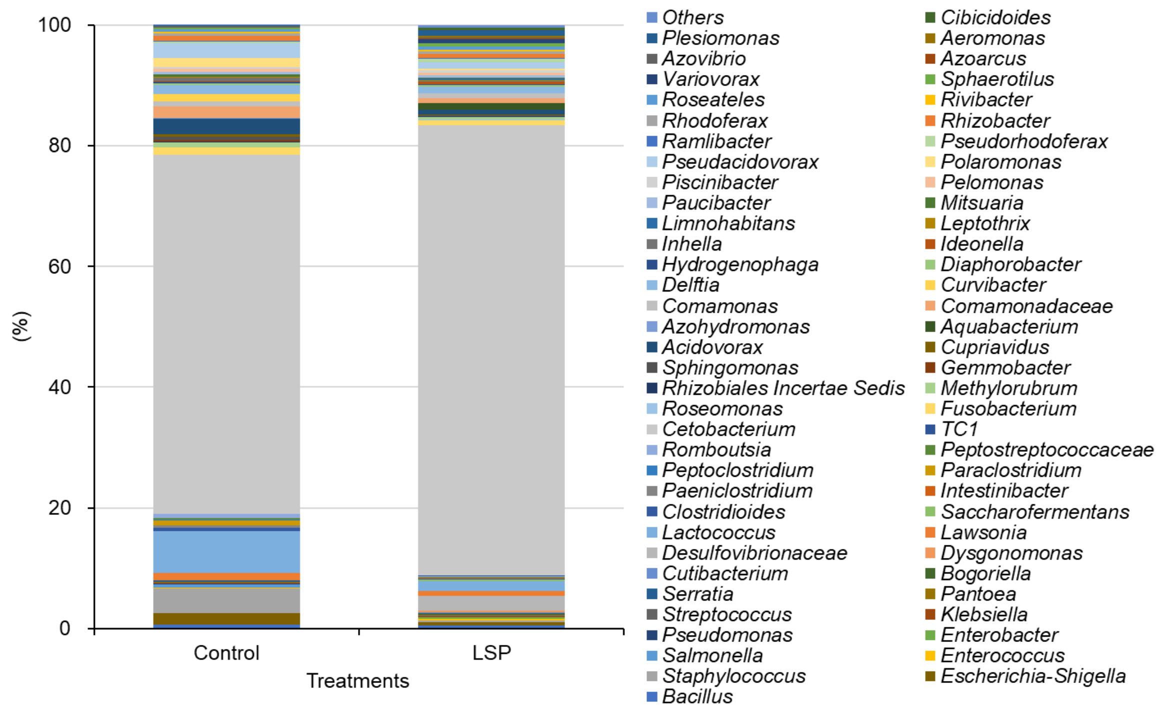

Figure S3. Relative abundance of major bacterial genera in the intestinal microbiota of Asian seabass fed a control diet without postbiotics or a low-dose postbiotic diet (LSP; 1×10^8 cells kg^{-1} diet) for 56 days. Relative abundance is expressed as the percentage of total bacterial sequences detected in each treatment group.

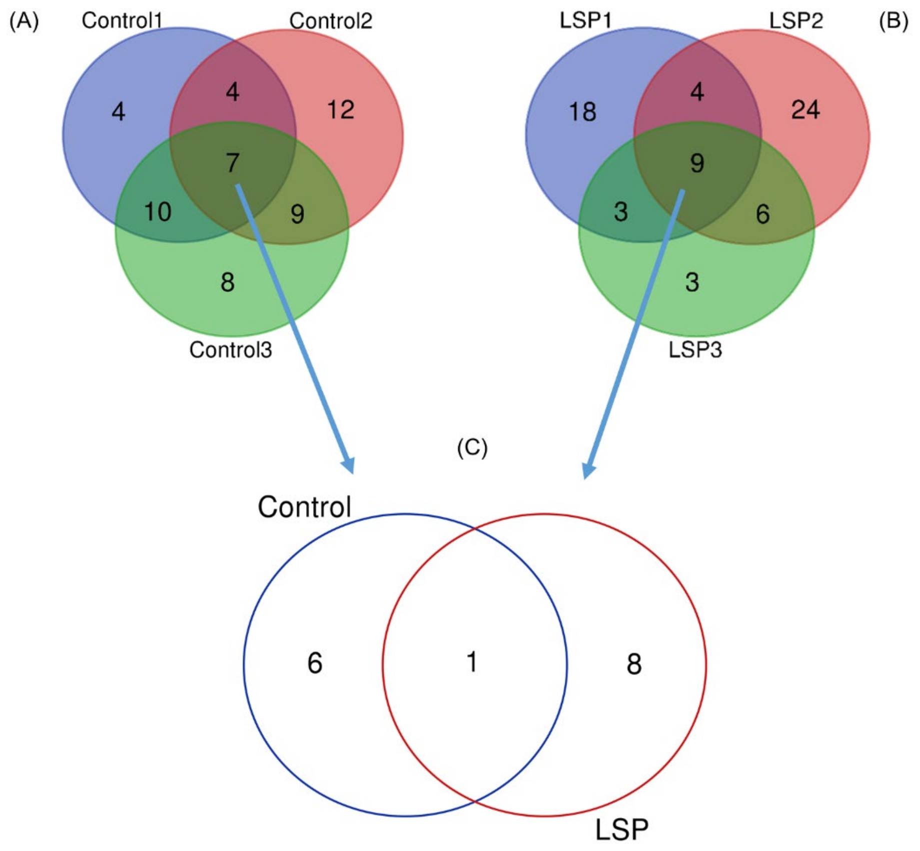

Figure S4. Venn diagram showing the shared and unique intestinal microbial genera detected in Asian seabass fed a control diet without postbiotics (A) or a low-dose postbiotic diet (LSP; 1×10^8 cells kg^{-1} diet) (B) for 56 days. The overlapping area indicates genera common to both treatment groups, whereas the non-overlapping areas indicate genera unique to each group (C). Numbers represent the total genera identified in each category.

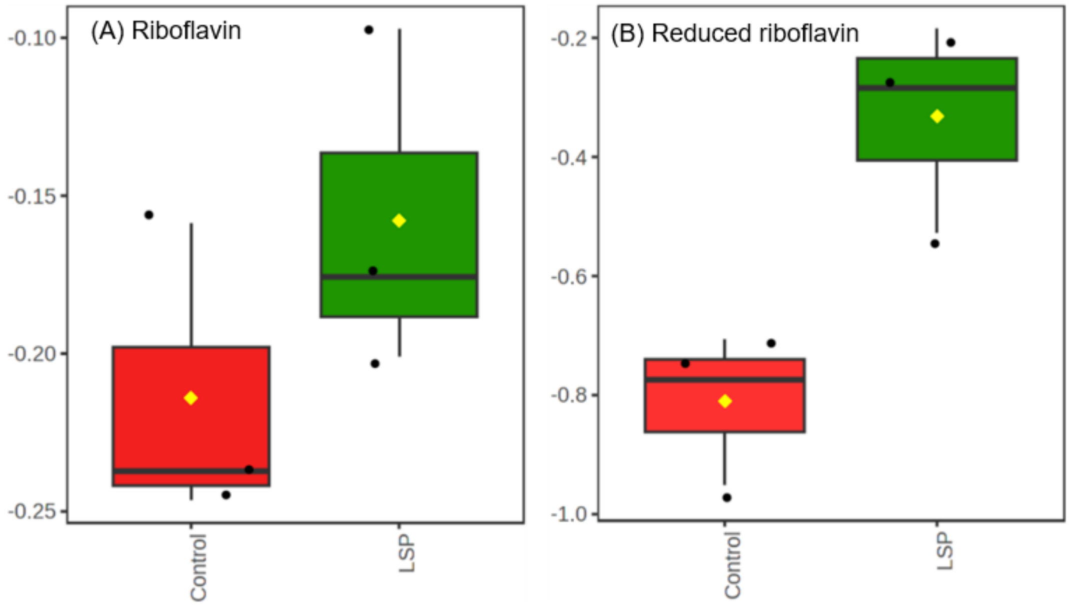

Figure S5. Relative abundances of riboflavin (A) and reduced riboflavin (B) in the liver of Asian seabass fed a control diet without postbiotics or a low-dose postbiotic diet (LSP; 1×10^8 cells kg^{-1} diet) for 56 days. Neither metabolite met the variable importance in projection (VIP) screening criterion of VIP > 1. The fold change (FC) and p value were 1.35 and 0.252 for riboflavin and 2.44 and 0.0186 for reduced riboflavin, respectively.
